# Supplementary material for: Yersinia actively downregulates type III secretion and adhesion at higher cell densities
Source: PLoS Pathog. 2025 Aug 12;21(8):e1013423. doi: 10.1371/journal.ppat.1013423 (PMC12404644; doi:10.1371/journal.ppat.1013423)
Supplement: S1 Fig — 150 min after the induction of T3SS assembly by temperature shift to 37° in secreting conditions, T3SS activation in a wild-type strain carrying the PyopE::sfGFP-ssrA reporter on the virulence plasmid was inhibited by adding 10 mM CaCl2 to the medium (t = 0). From t = 0, fluorescence was measured over time. n = 3, graph shows representative result. (PDF) [file ppat.1013423.s001.pdf]

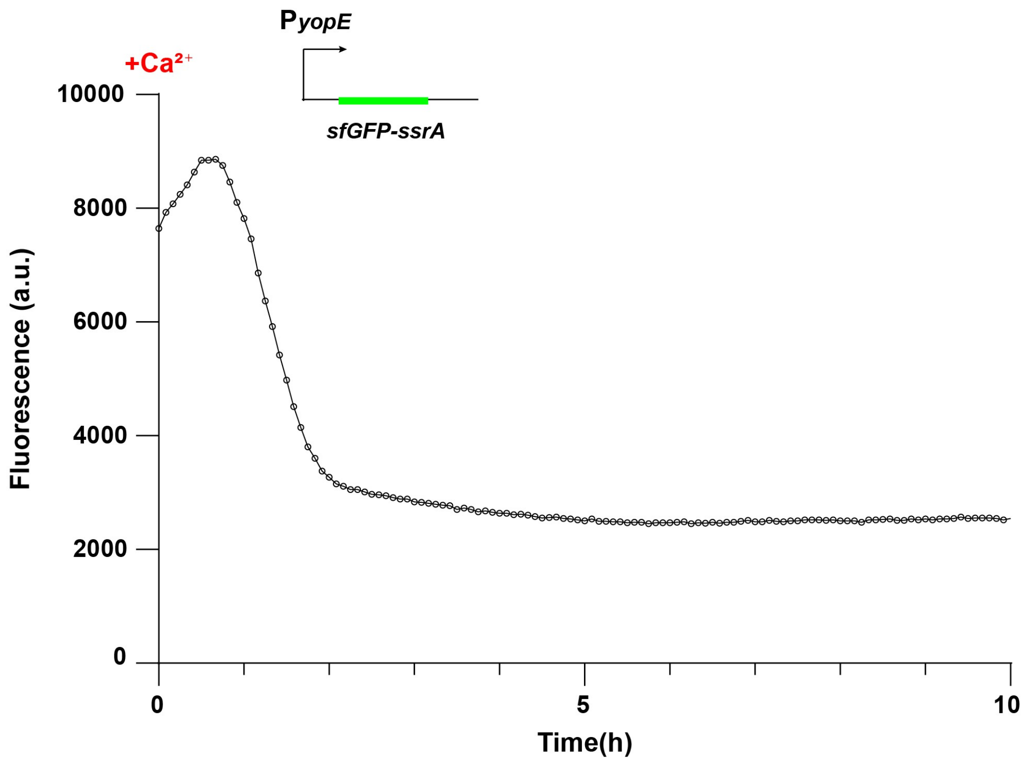

S1 Fig – Kinetics of the P_{yopE}::*sfGFP-ssrA* activity assay.

150 min after the induction of T3SS assembly by temperature shift to 37° in secreting conditions, T3SS activation in a wild-type strain carrying the P_{yopE}::*sfGFP-ssrA* reporter on the virulence plasmid was inhibited by adding 10 mM $CaCl_2$ to the medium (t=0). From t=0, fluorescence was measured over time. *n*=3, graph shows representative result.
