# Supplementary material for: Yersinia actively downregulates type III secretion and adhesion at higher cell densities
Source: PLoS Pathog. 2025 Aug 12;21(8):e1013423. doi: 10.1371/journal.ppat.1013423 (PMC12404644; doi:10.1371/journal.ppat.1013423)
Supplement: S2 Fig — Image of confocal microscopy sections (z = 0 µm, 13 µm, 27 µm) of a Y. enterocolitica ΔsctW PyopE-sfGFP-ssrA microcolony section at 37°. T3SS activity, which results in a strong upregulation of the yopE promoter and cellular fluorescence, is only detected at the edge of the microcolony. Scale bar, 50 µm; n = 3. (PDF) [file ppat.1013423.s002.pdf]

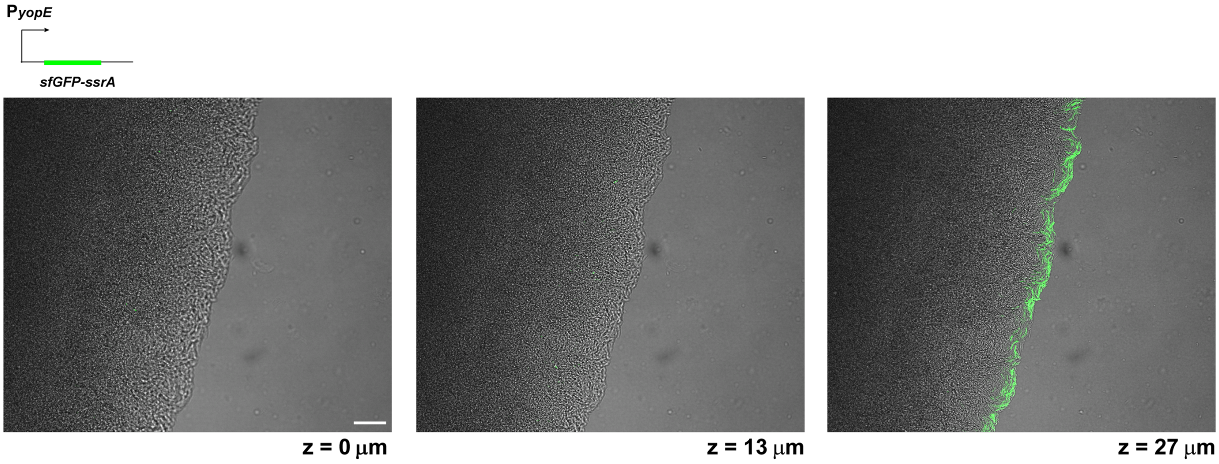


**S2 Fig – P$_{yopE}$::*sfGFP-ssrA* expression in *Y. enterocolitica* microcolonies.**

Image of confocal microscopy sections (z=0 µm, 13 µm, 27 µm) of a *Y. enterocolitica* Δ*sctW* P$_{yopE}$-*sfGFP-ssrA* microcolony section at 37°. T3SS activity, which results in a strong upregulation of the *yopE* promoter and cellular fluorescence, is only detected at the edge of the microcolony. Scale bar, 50 µm; *n*=3.
