# Supplementary material for: Yersinia actively downregulates type III secretion and adhesion at higher cell densities
Source: PLoS Pathog. 2025 Aug 12;21(8):e1013423. doi: 10.1371/journal.ppat.1013423 (PMC12404644; doi:10.1371/journal.ppat.1013423)
Supplement: S3 Fig — Optical density at 600 nm (OD600) of Y. enterocolitica wild-type cultures (MRS40) incubated at 28°C. n = 3; whiskers denote standard deviation. (PDF) [file ppat.1013423.s003.pdf]

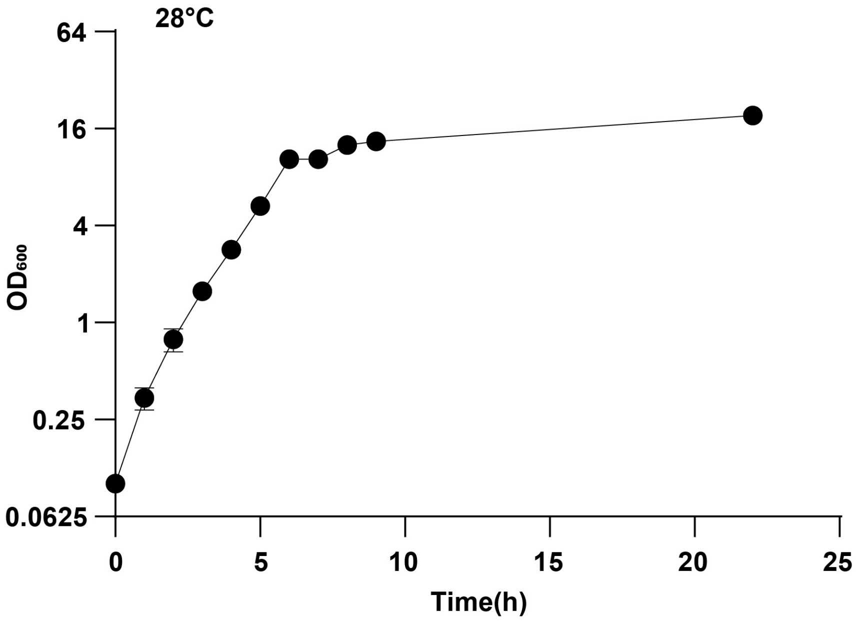

S3 Fig – *Y. enterocolitica* reference growth curve.

Optical density at 600 nm (OD_{600}) of *Y. enterocolitica* wild-type cultures (MRS40) incubated at 28°C. *n*=3; whiskers denote standard deviation.
