# Supplementary material for: Yersinia actively downregulates type III secretion and adhesion at higher cell densities
Source: PLoS Pathog. 2025 Aug 12;21(8):e1013423. doi: 10.1371/journal.ppat.1013423 (PMC12404644; doi:10.1371/journal.ppat.1013423)
Supplement: S4 Fig — T3SS reporter assay (PyopE-sfGFP-SsrA) of Yersinia cells inoculated at ODin=0.1 in secreting medium. Culture aliquots were shifted to 37°C to induce assembly of the T3SS at different time points post-inoculum as indicated. T3SS activity was measured at a single-cell level 2.5 h post-induction. n = 3 independent experiments; each dot represents a single-cell measurement and the black bar represents the average of the fluorescence intensity. (PDF) [file ppat.1013423.s004.pdf]

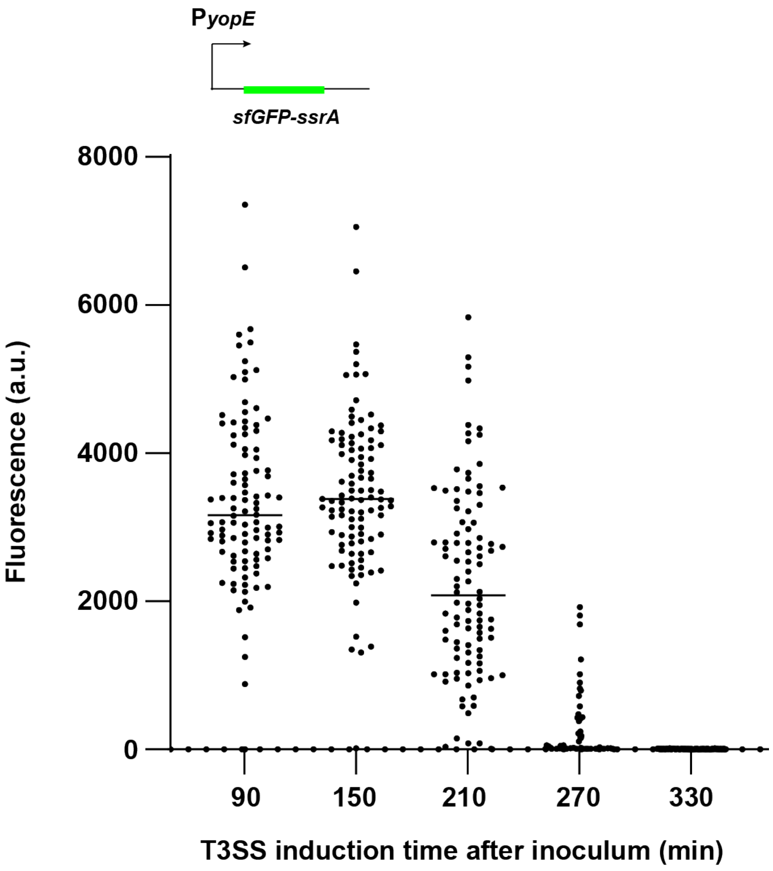

S4 Fig – T3SS activity is repressed over time in growing cultures.

T3SS reporter assay (P_{yopE}-*sfGFP-SsrA*) of *Yersinia* cells inoculated at OD_{in}=0.1 in secreting medium. Culture aliquots were shifted to 37°C to induce assembly of the T3SS at different time points post-inoculum as indicated. T3SS activity was measured at a single-cell level 2.5 h post-induction. *n*=3 independent experiments; each dot represents a single-cell measurement and the black bar represents the average of the fluorescence intensity.
