# Supplementary material for: Yersinia actively downregulates type III secretion and adhesion at higher cell densities
Source: PLoS Pathog. 2025 Aug 12;21(8):e1013423. doi: 10.1371/journal.ppat.1013423 (PMC12404644; doi:10.1371/journal.ppat.1013423)
Supplement: S5 Fig — OD600 of wild-type strain expressing PyopE::sfGFP-ssrA under secreting conditions at the different ODin used in Fig 2b (0.1, 0.3, 0.7, 1.0, 1.5). OD600 was measured from the time of the shift to 37° (t = 0). n = 3, whiskers denote standard deviation. (PDF) [file ppat.1013423.s005.pdf]

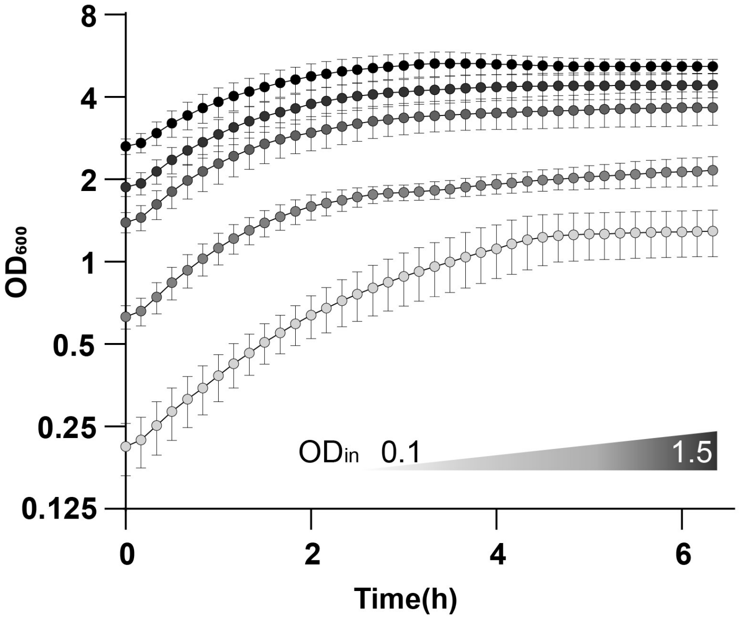

S5 Fig – *Y. enterocolitica* growth curves at different OD_{in}.

OD_{600} of wild-type strain expressing P_{yopE}*::sfGFP-ssrA* under secreting conditions at the different OD_{in} used in Fig 2b (0.1, 0.3, 0.7, 1.0, 1.5). OD_{600} was measured from the time of the shift to 37° (t=0). *n*=3, whiskers denote standard deviation.
