# Supplementary material for: Yersinia actively downregulates type III secretion and adhesion at higher cell densities
Source: PLoS Pathog. 2025 Aug 12;21(8):e1013423. doi: 10.1371/journal.ppat.1013423 (PMC12404644; doi:10.1371/journal.ppat.1013423)
Supplement: S6 Fig — Volcano plot showing differences in the expression of Y. enterocolitica proteins between cultures grown under secreting conditions at ODin 1.5 and 0.1. All proteins with at least three detected peptides are displayed. Representation of the plot shown in Fig 3a (Table 1, S2 Table), highlighting different classes of proteins, colored according to their function. (PDF) [file ppat.1013423.s006.pdf]

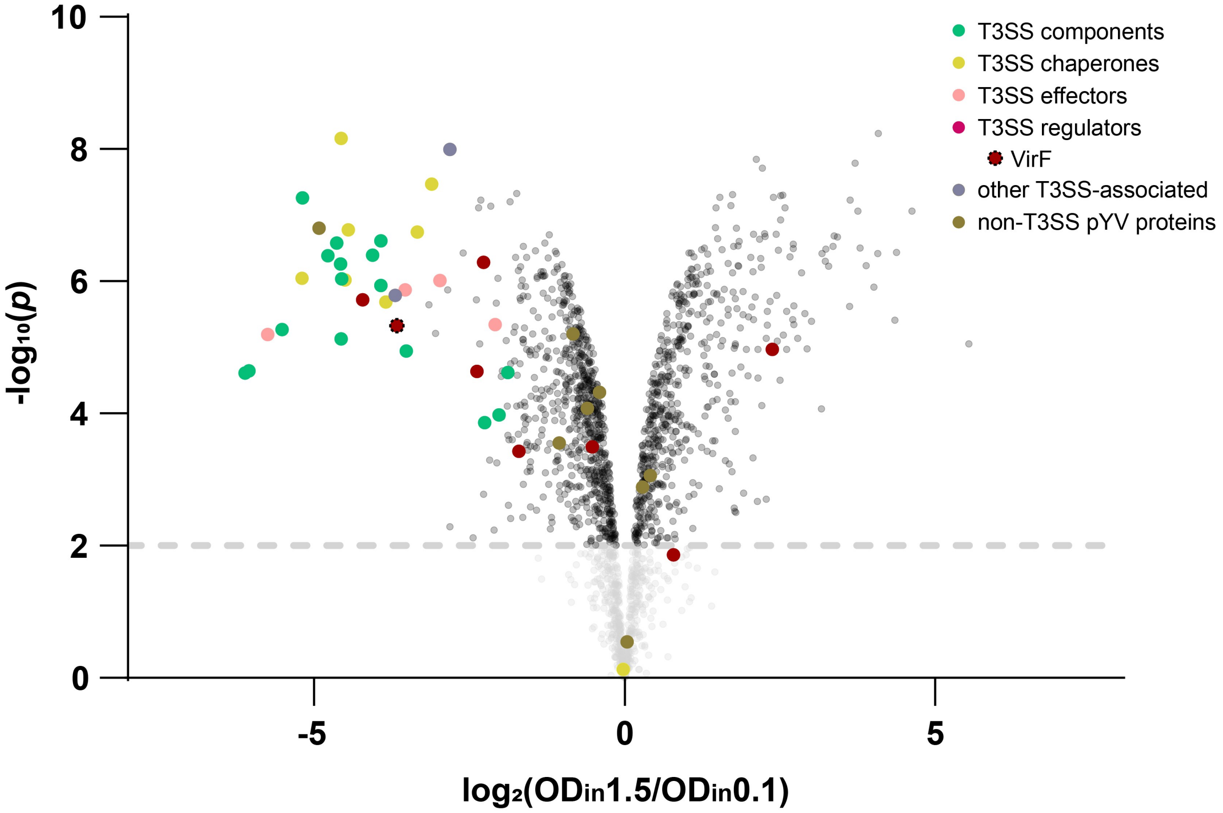

S6 Fig – All T3SS gene categories are similarly downregulated at higher cell densities.

Volcano plot showing differences in the expression of *Y. enterocolitica* proteins between cultures grown under secreting conditions at OD_{in} 1.5 and 0.1. All proteins with at least three detected peptides are displayed. Representation of the plot shown in Fig 3a (Table 1, Suppl. Table 2), highlighting different classes of proteins, colored according to their function.
