# Supplementary material for: Yersinia actively downregulates type III secretion and adhesion at higher cell densities
Source: PLoS Pathog. 2025 Aug 12;21(8):e1013423. doi: 10.1371/journal.ppat.1013423 (PMC12404644; doi:10.1371/journal.ppat.1013423)
Supplement: S7 Fig — Gel band intensity quantification of secretion assay of wild-type (WT, light blue) and quorum sensing deletion strain (ΔQS = ΔyenI, ΔlsrK, dotted bars) at ODin = 0.1, 0.3, 0.7, 1.0, 1.5 shown in Fig 4a. n = 4, each spot represents a single measurement, whiskers denote standard deviation. For statistics, WT and ΔQS measurements were compared for each ODin using an unpaired t-test. Each comparison resulted in a statistically non-significant difference (ns, p > 0.05), represented in the graph as grouped result. (PDF) [file ppat.1013423.s007.pdf]

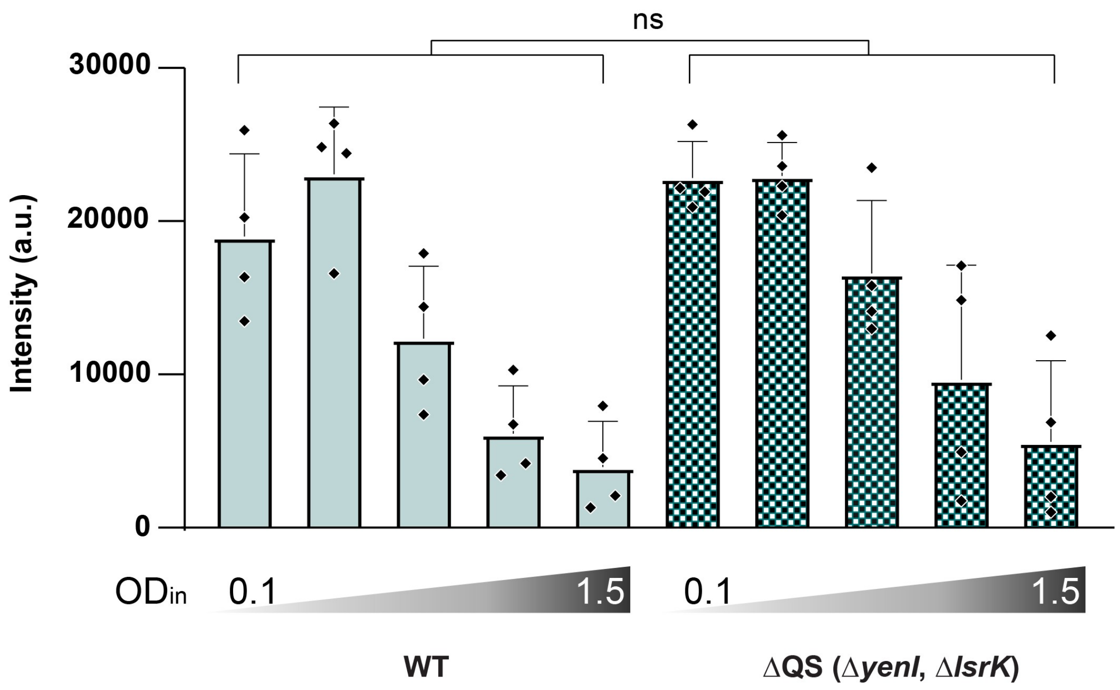

S7 Fig – The density-dependent downregulation of the T3SS is not conferred by quorum sensing.

Gel band intensity quantification of secretion assay of wild-type (WT, light blue) and quorum sensing deletion strain (ΔQS = *ΔyenI, ΔlsrK*, dotted bars) at OD_{in} = 0.1, 0.3, 0.7, 1.0, 1.5 shown in Fig.4a. n=4, each spot represents a single measurement, whiskers denote standard deviation. For statistics, WT and ΔQS measurements were compared for each OD_{in} using an unpaired t-test. Each comparison resulted in a statistically non-significant difference (ns, p>0.05), represented in the graph as grouped result.
