# Supplementary material for: Yersinia actively downregulates type III secretion and adhesion at higher cell densities
Source: PLoS Pathog. 2025 Aug 12;21(8):e1013423. doi: 10.1371/journal.ppat.1013423 (PMC12404644; doi:10.1371/journal.ppat.1013423)
Supplement: S8 Fig — T3SS reporter assay (PyopE-sfGFP-SsrA) of Yersinia cells at ODin=0.1 and 1.5 in the indicated strains after shifting the culture to 37°C (t = 0), which induces the expression of the T3SS. n = 3, shadowed area denotes standard deviation. (PDF) [file ppat.1013423.s008.pdf]

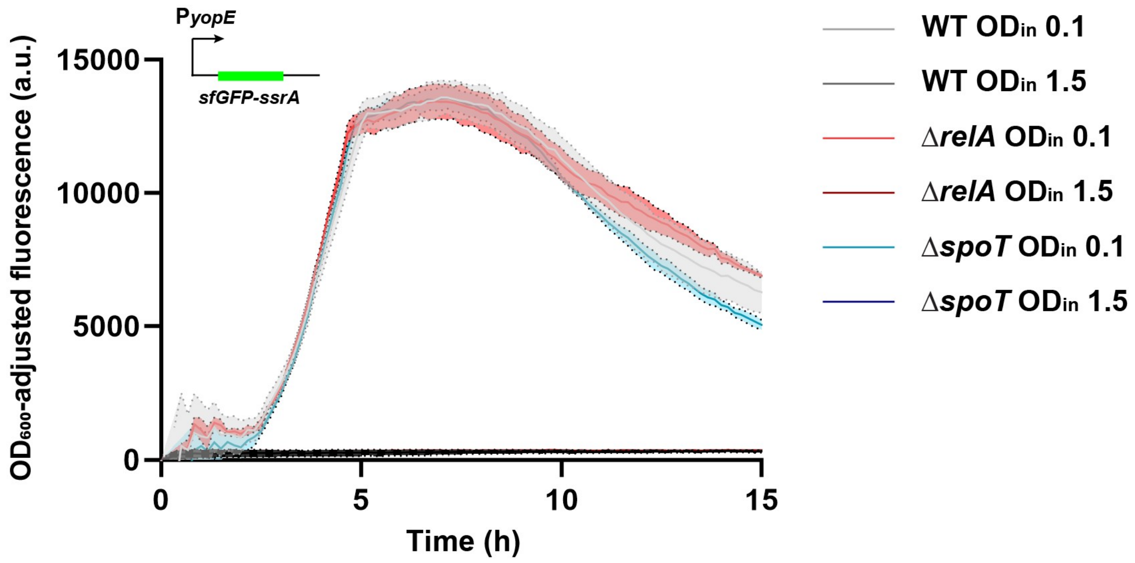

S8 Fig – The stringent response regulators RelA and SpoT do not significantly contribute to the density-dependent downregulation of the T3SS.

T3SS reporter assay (P_{yopE}-*sfGFP-SsrA*) of *Yersinia* cells at OD_{in}=0.1 and 1.5 in the indicated strains after shifting the culture to 37°C (t=0), which induces the expression of the T3SS. *n*=3, shadowed area denotes standard deviation.
