# Supplementary material for: Yersinia actively downregulates type III secretion and adhesion at higher cell densities
Source: PLoS Pathog. 2025 Aug 12;21(8):e1013423. doi: 10.1371/journal.ppat.1013423 (PMC12404644; doi:10.1371/journal.ppat.1013423)
Supplement: S9 Fig — a) Schematic display of the genes encoding for T3SS structural components (operons virA, virB, virC, and translocator operon (transl.), dark grey), T3SS effectors and chaperones (lighter shades of grey), and VirF (purple) on the pYV virulence plasmid (left), and the principle working mechanism of VirF (right). b) Secretion assay displaying the effectors secreted by the T3SS at ODin = 0.1 in wild-type (WT) and ΔvirF background. Lanes 2–6, increasing expression of VirF in trans (arabinose concentrations of 0, 0.001, 0.003, 0.008%, respectively). n = 3, gel shows representative result. (PDF) [file ppat.1013423.s009.pdf]

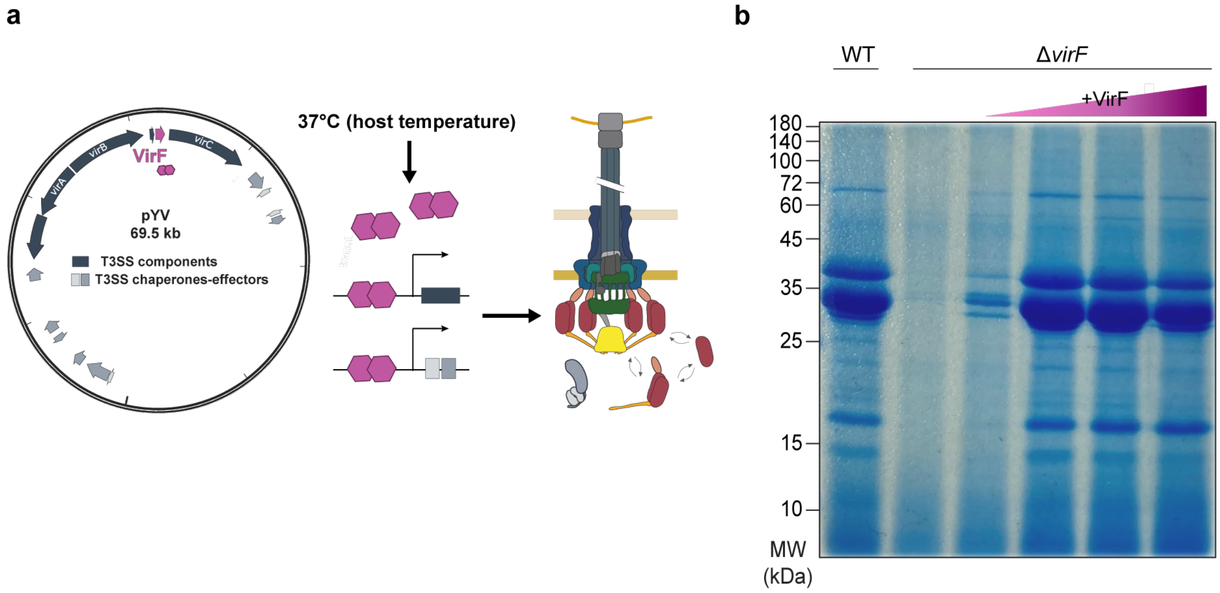


**S9 Fig – VirF is essential for T3SS secretion.**

**a)** Schematic display of the genes encoding for T3SS structural components (operons *virA*, *virB*, *virC*, and translocator operon (transl.), dark grey), T3SS effectors and chaperones (lighter shades of grey), and VirF (purple) on the pYV virulence plasmid (left), and the principle working mechanism of VirF (right). Image based on a plasmid map created by the SnapGene software (GSL Biotech). **b)** Secretion assay displaying the effectors secreted by the T3SS at $OD_{in}$ = 0.1 in wild-type (WT) and Δ*virF* background. Lanes 2-6, increasing expression of VirF *in trans* (arabinose concentrations of 0, 0.001, 0.003, 0.008%, respectively). *n*=3, gel shows representative result.
