# Supplementary material for: Yersinia actively downregulates type III secretion and adhesion at higher cell densities
Source: PLoS Pathog. 2025 Aug 12;21(8):e1013423. doi: 10.1371/journal.ppat.1013423 (PMC12404644; doi:10.1371/journal.ppat.1013423)
Supplement: S10 Fig — Image of confocal microscopy sections (z = 0 µm, 13 µm, 27 µm) of a Y. enterocolitica ΔsctW PyopE-sfGFP-ssrA microcolony additionally expressing VirF (induced by 0.2% arabinose) at 37°C. T3SS activity, visualized by the sfGFP signal, is detected throughout the colony. Scale bar, 50 µm, n = 3. (PDF) [file ppat.1013423.s010.pdf]

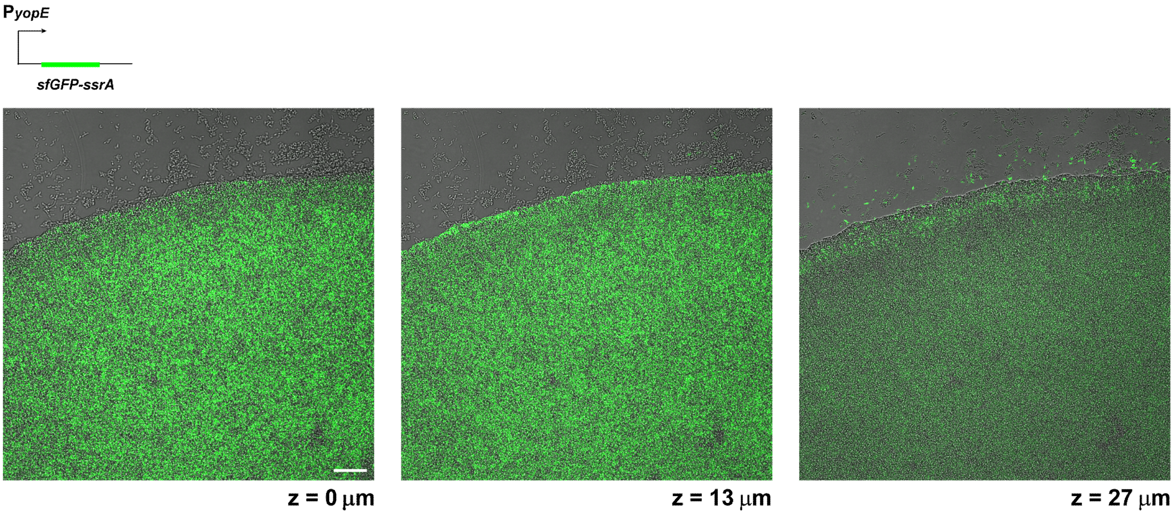

S10 Fig – Additional expression of the transcriptional regulator VirF restores T3SS secretion in a *Yersinia* microcolony.

Image of confocal microscopy sections (z=0 µm, 13 µm, 27 µm) of a *Y. enterocolitica* Δ*sctW* P_{yopE}-*sfGFP-ssrA* microcolony additionally expressing VirF (induced by 0.2% arabinose) at 37°C. T3SS activity, visualized by the sfGFP signal, is detected throughout the colony. Scale bar, 50 µm, *n*=3.
