# Supplementary material for: Yersinia actively downregulates type III secretion and adhesion at higher cell densities
Source: PLoS Pathog. 2025 Aug 12;21(8):e1013423. doi: 10.1371/journal.ppat.1013423 (PMC12404644; doi:10.1371/journal.ppat.1013423)
Supplement: S11 Fig — Expression of mCherry-SctL (expected molecular weight 51.4 kDa) and SctG-sfGFP (expected molecular weight 41.5 kDa) expressed from the native genetic location in Y. enterocolitica in secreting medium at the ODin indicated. Immunoblot using antibodies directed against mCherry and EGFP as indicated. Control: Expression of mCherry (expected size 26.7 kDa) and GFP (expected size 27.0 kDa) from plasmid. n = 3, blots show representative results. (PDF) [file ppat.1013423.s011.pdf]

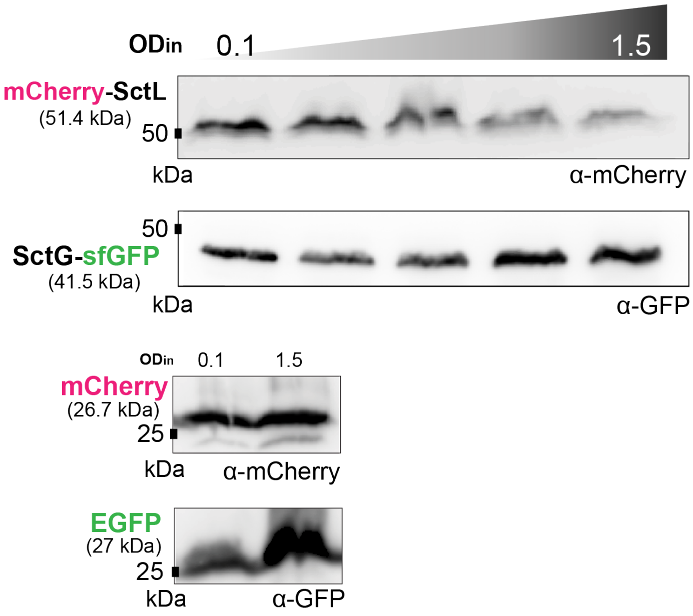

S11 Fig – SctG protein levels are stable at higher local cell densities.

Expression of mCherry-SctL (expected molecular weight 51.4 kDa) and SctG-sfGFP (expected molecular weight 41.5 kDa) expressed from the native genetic location in *Y. enterocolitica* in secreting medium at the OD_{in} indicated. Immunoblot using antibodies directed against mCherry and EGFP as indicated. Control: Expression of mCherry (expected size 26.7 kDa) and GFP (expected size 27.0 kDa) from plasmid. *n*=3, blots show representative results.
