# Supplementary material for: Yersinia actively downregulates type III secretion and adhesion at higher cell densities
Source: PLoS Pathog. 2025 Aug 12;21(8):e1013423. doi: 10.1371/journal.ppat.1013423 (PMC12404644; doi:10.1371/journal.ppat.1013423)
Supplement: S12 Fig — DNA sequence alignment using Clustal Omega. Nucleotides that are identical in both sequences are indicated by asterisks. (PDF) [file ppat.1013423.s012.pdf]

```
CsrB Y. enterocolitica          GCCCGATAGGATCTGGC-GGAAAGGACGGTATCAGGATGGTGCCACTTCAGGATGAAGGA      59
CsrB Y. pseudotuberculosis      GCTGGATAGGATCTGGCGGAGAGGGAACGCATCTGGAAGATGTGTCTTCAGGACGAAGAA      60
                                **  ************* *  * ***  * *** *** * **   ******** **** *

CsrB Y. enterocolitica          CTCAGGGACTGCTTAGGATGAGTGAAGGGATGTTTCAGGAAGAAACAAAGGACACCTCCA      119
CsrB Y. pseudotuberculosis      CACAGGGACTGCTTAGGACGAGTGAAGGGACGTTTCAGGATGAAACAAGGGACACCTCCA      120
                                * **************** *********** ********* ******* ***********

CsrB Y. enterocolitica          GGATGGAGATTGAGAGCCAGTTCAGGATGATTGGTGGGTTAGGATAACTTCAGGATTGGC      179
CsrB Y. pseudotuberculosis      GGATGGAGATTGAGAGCCAGTTCAGGATGATTGGTGGGTTAGGATAGCCTAAGGATTAAC      180
                                ********************************************** * * ******  *

CsrB Y. enterocolitica          ACTGGGATGGTGTAGGACAACGCGACGGATTGCTGGTTAGGATAACCGCACGGAAAAGTT      239
CsrB Y. pseudotuberculosis      GCCGGGATGGTGTATAACATTGCGATGGATTGCTGGTTAGTATAACCATACGGAAAAGTT      240
                                 * ***********  ***  **** ************** ******  ***********

CsrB Y. enterocolitica          TTCAAGGATTGAGCAGGGAGCATCACTTTTAGCTGGATTGCTATGAAACGAATAGAGGGG      299
CsrB Y. pseudotuberculosis      TTCAGGGATTGAGCAGGGAGCATCAATTTTAGCTGGATTGCTATAAAACGAATTGAGGGG      300
                                **** ******************** ****************** ******** ******

CsrB Y. enterocolitica          TACTGGTAAAACAGTACCCCTTTT  323
CsrB Y. pseudotuberculosis      TACTGGTAAAACAGTACCCCTTTT  324
                                ************************


CsrC Y. enterocolitica          TGACTATTTTTTGTAACTCATGGTTTTTTAACAGTGCGGGATGTACTGGC---AAGGAGC      57
CsrC Y. pseudotuberculosis      ----------------------ATACAAGGAATGTAATGGATGTACGGGAGCCAAGGACG      38
                                                       *      *  **   ******** **    *****

CsrC Y. enterocolitica          GTAATCACTTAGGAAGAGTGGGGTATGCTTAAGGAATGTAATGGATATACTGTGAGCCAG      117
CsrC Y. pseudotuberculosis      CTATAAACGGAG-----CTAATGTAAAGGGATGTTAGGACACTGGCCGGAGCGCCGGGCG      93
                                 **   **  **      *   ***     * *  * *  *  *           *   *

CsrC Y. enterocolitica          GGACACCTTCAGGGTTGGGGGGATGGCAAGGATGGCGAATTGCAGTAGGGAGAAACCGGG      177
CsrC Y. pseudotuberculosis      AATCGCCTTCAGGGTTTGAGGGGTGGCAAGGATCGCGTATTGCAGGAGGGAGAAACCGGG      153
                                   * *********** * *** ********** *** ******* **************

CsrC Y. enterocolitica          ACGTTATATCAGTGTAGGGAATGCACAATAAGGATATCCTTCCGCAGAAGGTGCGAAAAA      237
CsrC Y. pseudotuberculosis      ACGTTATCTAAGTGCAGGGAGTGCGCTGTAAGGATATCCTTCCGCAGAAGGTGCGAAAAA      213
                                ******* * **** ***** *** *  ********************************

CsrC Y. enterocolitica          AGGCGACAGGTTAATCTGCCGCCTTTTTTCTTTCTCTTCTT   278
CsrC Y. pseudotuberculosis      AGGCGACAGGTTAACCTGCCGCCTTTTTTCTTTCTCTTCTT   254
                                ************** **************************
```

S12 Fig – Sequence conservation of CsrB and CsrC in *Y. enterocolitica* and *Y. pseudo-tuberculosis*.

DNA sequence alignment using Clustal Omega. Nucleotides that are identical in both sequences are indicated by asterisks.
