# Supplementary material for: Yersinia actively downregulates type III secretion and adhesion at higher cell densities
Source: PLoS Pathog. 2025 Aug 12;21(8):e1013423. doi: 10.1371/journal.ppat.1013423 (PMC12404644; doi:10.1371/journal.ppat.1013423)
Supplement: S13 Fig — Relative fluorescence of a pBAD::virF(-123/ + 75)-sfGFP reporter strain at ODin 0.1, compared to an identically treated pBAD::sfGFP control, measured in technical triplicates in a 96-well-plate after shifting the cultures to 37°C (t = 0), which induces the expression of the T3SS. Fluorescence is reduced in a (-16/-14: GGA to TTC) mutant (orange) with reduced CsrA binding [55] and at higher densities (ODin 1.5, open circles). n = 3 biological replicates. (PDF) [file ppat.1013423.s013.pdf]

```
Y.ent.    GTGATTTATTATATTGGTTTTGGTTGCATTAATCGATGGTTGTACATCGCACGCATAATA  31992
Y.ptb.    GTGATTTATTATATTGGTTTTGGTTGCATTAATCGATGGTTGTACATCGCACGCATAATA  51224
          ************************************************************

Y.ent.    ACTCAATACACCTCATTAGATAAATATATACAAGTTTTAGATTTTTAGGACAGTATAACA  32052
Y.ptb.    ACTCAATACACCTCATTAGATAAATATATACAAGTTTTAGATTTTTAGGACAGTATAACA  51284
          ************************************************************

Y.ent.    TTTATGGCATCACTAGAGATTATTAAATTAGAATGGGCCACACCTATATTTAAGGTTGTT  32112
Y.ptb.    TTTATGGCATCACTAGAGATTATTAAATTAGAATGGGTCACACCTATATTTAAGGTTGTT  51344
          ************************************ ***********************

Y.ent.    GAGCATTCACAAGATGGCCTATATATTCTTTTGCAAGGTCAGATTTCATGGCAGAACAGC  32172
Y.ptb.    GAGCATTCACAAGATGGCCTATATATTCTTTTGCAAGGTCAGATTTCATGGCAGAGCAGC  51404
          ******************************************************* ****

Y.ent.    AGTCAGACATATGATTTAGATGAGGGGAATATGCTGTTTTTGCGTCGTGGCAGCTATGCT  32232
Y.ptb.    GGTCAGACATATGATTTAGATGAGGGGAATATGCTGTTTTTGCGTCGTGGCAGCTATGCT  51464
           ***********************************************************

Y.ent.    GTTCGATGTGGTACAAAAGAACCCTGCCAATTACTTTGGATTCCATTACCAGGCAGTTTT  32292
Y.ptb.    GTTCGATGTGGTACAAAAGAACCCTGCCAATTACTTTGGATTCCATTACCCGGCAGTTTT  51524
          ************************************************** *********

Y.ent.    TTGAGTACTTTTTTACATCGGTTTGGTTCTTTGCTTAGTGAAATTAGACGAGACAATGCC  32352
Y.ptb.    TTGAGTACTTTTTTGCATCGCTTTGGTTCTTTGCTTAGTGAAATTGGACGAGACAACTCC  51584
          ************** ***** ************************ **********  **

Y.ent.    ACACCCAAGCCATTGTTAATTTTTAATATTTCACCAATATTATCACAATCCATTCAAAAT  32412
Y.ptb.    ACACCCAAACCATTGTTAATTTTTAATATTTCACCAATATTATCACAATCCATTCAAAAT  51644
          ******** ***************************************************

Y.ent.    CTATGTGCCATATTGGAACGGAGTGATTTTCCGTCAGTATTAACGCAACTGCGTATTGAG  32472
Y.ptb.    CTATGTGCCATATTGGAACGGAGTGATTTTCCGTCAGTATTAACGCAACTGCGTATTGAG  51704
          ************************************************************

Y.ent.    GAATTATTGCTTTTGCTTGCCTTTAGCTCGCAAGGGGCTTTATTCCTCTCGGCTCTGCGC  32532
Y.ptb.    GAATTACTGCTTTTGCTTGCCTTTAGCTCGCAAGGGACTTTATTTCTCTCGGCTCTGCGC  51764
          ****** ***************************** ******* ***************

Y.ent.    CATTTAGGCAACCGCCCAGAAGAACGGTTGCAGAAATTTATGGAGGAAAATTATCTACAA  32592
Y.ptb.    CATTTAGGCAATCGCCCAGAAGAACGGTTGCAAAAATTTATGGAGGAAAATTATCTACAA  51824
          *********** ********************* **************************

Y.ent.    GGGTGGAAACTAAGCAAATTTGCGCGAGAATTCGGCATGGGATTAACCACATTCAAAGAA  32652
Y.ptb.    GGGTGGAAGCTAAGCAAATTTGCGCGAGAATTCGGCATGGGATTAACCACATTCAAAGAA  51884
          ******** ***************************************************

Y.ent.    CTGTTTGGTACAGTTTATGGCATTTCACCACGCGCCTGGATAAGCGAGCGACGTATTCTC  32712
Y.ptb.    CTGTTTGGTACAGTTTATGGCATTTCACCACGCGCCTGGATAAGCGAGCGACGTATTCTC  51944
          ************************************************************

Y.ent.    TATGCTCACCAATTACTTCTTAATGGTAAGATGAGTATTGTTGATATTGCCATGGAAGCA  32772
Y.ptb.    TATGCTCACCAATTACTTCTTAATGGTAAGATGAGTATTGTTGATATTGCCATGGAAGCG  52004
          ***********************************************************

Y.ent.    GGGTTCTCGAGTCAGTCTTATTTCACTCAAAGTTATCGACGTCGCTTCGGATGCACTCCC  32832
Y.ptb.    GGGTTCTCGAGTCAGTCTTATTTCACTCAAAGTTATCGACGTCGCTTCGGATGCACTCCA  52064
          ***********************************************************

Y.ent.    AGCCAAGCCCGTCTTACTAAAATAGCAACCACAGGCTAA  32871
Y.ptb.    AGCCAAGCCCGTCTTACTAAAATAGCAACCACAGGCTAA  52103
          ***************************************
```

S13 Fig - Sequence conservation of the *virF*/*lcrF* upstream intergenic region and coding region in *Y. enterocolitica* and *Y. pseudotuberculosis*.

DNA sequence alignment using BLAST for the *sctG*-*virF* intergenic region and coding region of *Yersinia enterocolitica* (*Y.ent.*, virulence plasmid pYVe227, NCBI accession AF102990.1, nucl. 31933-32871) and *Y. pseudotuberculosis* (*Y.ptb.*, virulence plasmid pIB1, NCBI accession NZ_CP032567.1, nucl. 51165 – 52103). 921 of the 939 nucleotides (98%) are identical. VirF start and stop codons are marked in blue, bold and italic font, respectively.
