# Supplementary material for: Yersinia actively downregulates type III secretion and adhesion at higher cell densities
Source: PLoS Pathog. 2025 Aug 12;21(8):e1013423. doi: 10.1371/journal.ppat.1013423 (PMC12404644; doi:10.1371/journal.ppat.1013423)
Supplement: S14 Fig — a) Changes in the transcript levels of CsrB and CsrC at ODin=1.5 upon CsrA overexpression (CsrA+, 0.2% arabinose) in comparison to the wildtype strain (WT), as measured by reverse transcription quantitative PCR. Additional CsrA expression is compensated by higher levels of CsrB and, to a lower degree, CsrC. b) T3SS reporter assay (PyopE-sfGFP-ssrA) at ODin 0.1 and 1.5 in the indicated strains after shifting the culture to 37°C (t = 0), which induces the expression of the T3SS. n = 3 for all panels; shadowed area in b) denotes standard deviation. (PDF) [file ppat.1013423.s014.pdf]

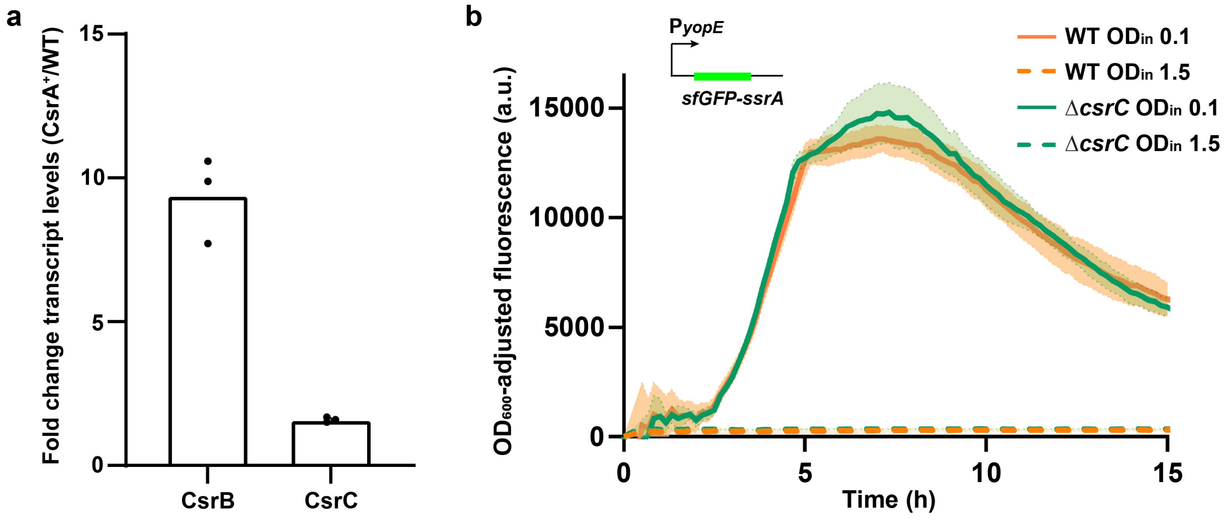

S14 Fig – Manipulations of the CsrABC system are efficiently compensated.

a) Changes in the transcript levels of CsrB and CsrC at OD_{in}=1.5 upon CsrA overexpression ($CsrA^+$, 0.2% arabinose) in comparison to the wildtype strain (WT), as measured by reverse transcription quantitative PCR. Additional CsrA expression is compensated by higher levels of CsrB and, to a lower degree, CsrC. **b)** T3SS reporter assay (P_{yopE}-*sfGFP-ssrA*) at OD_{in} 0.1 and 1.5 in the indicated strains after shifting the culture to 37°C (t=0), which induces the expression of the T3SS. *n*=3 for all panels; shadowed area in b) denotes standard deviation; values of both OD_{in}=1.5 cultures overlap near x axis.
