# Supplementary material for: Yersinia actively downregulates type III secretion and adhesion at higher cell densities
Source: PLoS Pathog. 2025 Aug 12;21(8):e1013423. doi: 10.1371/journal.ppat.1013423 (PMC12404644; doi:10.1371/journal.ppat.1013423)
Supplement: S1 Table — Quantification of EGFP-SctQ foci per bacterium, corresponding to assembled injectisomes, visualized by fluorescence microscopy at the indicated ODin values. Corresponds to Fig 2c. (PDF) [file ppat.1013423.s015.pdf]

S1 Table – Quantification of EGFP-SctQ foci per bacterium at different bacterial densities.

Quantification of EGFP-SctQ foci per bacterium, corresponding to assembled injectisomes, visualized by fluorescence microscopy at the indicated OD_{in} values. Corresponds to Fig 2c.

OD_{in}	0.1	0.3	0.7	1.0	1.5
# bacteria	369	369	369	369	369
0 foci	39	131	288	350	364
1 focus	92	103	59	16	5
2 foci	70	59	16	1	0
3 foci	76	45	5	1	0
4 foci	61	19	1	0	0
5 foci	20	6	0	0	0
6 foci	8	5	0	0	0
7 foci	3	1	0	0	0
≥8 foci	0	0	0	1	0
