# Supplementary material for: Yersinia actively downregulates type III secretion and adhesion at higher cell densities
Source: PLoS Pathog. 2025 Aug 12;21(8):e1013423. doi: 10.1371/journal.ppat.1013423 (PMC12404644; doi:10.1371/journal.ppat.1013423)
Supplement: S2 Table — While the adhesin YadA is downregulated similarly to the T3SS components (Table 1), most non-T3SS proteins encoded on the Yersinia pYV virulence plasmid are not strongly affected by the density-dependent downregulation. Label-free quantitative mass spectrometry in the total proteome of a ΔHOPEMTasd wild-type strain at the different growth conditions indicated, experiment and display format as shown in Table 1. (PDF) [file ppat.1013423.s016.pdf]

S2 Table – Density-dependent regulation of expression of non-T3SS components encoded on the *Yersinia* virulence plasmid.

While the adhesin YadA is downregulated similarly to the T3SS components (Table 1), most non-T3SS proteins encoded on the *Yersinia* pYV virulence plasmid are not strongly affected by the density-dependent downregulation. Label-free quantitative mass spectrometry in the total proteome of a ΔHOPEMTasd wild-type strain at the different growth conditions indicated, experiment and display format as shown in Table 1.

Protein	Log_2 intensity ratio	*p* value	Individual replicate log_2 intensity values						# pept.
			OD_{in} = 0.1			OD_{in} = 1.5			
Adhesin YadA	-4.94	1.6E-07	33.42	33.44	33.51	28.23	28.61	28.72	65
Resolvase TnpR	-1.26	3.6E-04	24.11	24.09	24.14	22.50	23.04	23.00	6
Antitoxin ParD	-0.83	6.3E-06	28.76	28.79	28.83	27.98	28.01	27.90	14
Toxin ParE	-0.60	8.3E-05	25.93	25.88	25.93	25.37	25.22	25.36	4
Transposase TnpA	-0.43	2.8E-03	22.55	22.25	22.41	21.97	21.96	21.99	2
Plasmid partitioning protein SpyA	-0.40	4.8E-05	28.32	28.33	28.35	27.94	27.92	27.94	21
Plasmid partitioning protein SpyB	0.04	2.8E-01	28.43	28.39	28.38	28.46	28.46	28.42	20
Arsenate reductase ArsC	0.29	1.3E-03	26.37	26.38	26.29	26.66	26.65	26.58	14
Arsenic resistance protein ArsH	0.42	8.7E-04	25.51	25.34	25.40	25.77	25.89	25.83	10
Arsenite inducible repressor ArsR	0.79	6.9E-03	20.40	20.89	21.07	21.49	21.71	21.55	3
