# Supplementary material for: Yersinia actively downregulates type III secretion and adhesion at higher cell densities
Source: PLoS Pathog. 2025 Aug 12;21(8):e1013423. doi: 10.1371/journal.ppat.1013423 (PMC12404644; doi:10.1371/journal.ppat.1013423)
Supplement: S3 Table — Label-free quantitative mass spectrometry of the known stationary response proteins CpxP, IclR and OsmY [a–d] in the total proteome of different growth conditions, as indicated. Stat., stationary cultures. # pept., number of total detected peptides. Display format as shown in Table 1 and S2 Table. (PDF) [file ppat.1013423.s017.pdf]

S3 Table – The density-dependent downregulation of the T3SS differs from stationary phase response.

Label-free quantitative mass spectrometry of the known stationary response proteins CpxP, IclR and OsmY [a-d] in the total proteome of different growth conditions, as indicated. Stat., stationary cultures. # pept., number of total detected peptides. Display format as shown in Table 1 and Suppl. Table 2.

Protein *(gene name)*	Log_2 intensity ratios		Individual replicate log_2 intensity values									# pept.
	OD_{in} 1.5 / OD_{in} 0.1	Stat. / OD_{in} 1.5	OD_{in} = 0.1			OD_{in} = 1.5			Stationary			
Osmotically-inducible protein Y *(osmY)*	0.77	1.63	26.23	26.17	26.22	26.92	27.00	27.03	28.65	28.55	28.65	16
Acetate operon repressor *(iclR)*	-0.29	2.02	23.66	23.41	23.60	23.29	23.31	23.22	25.19	25.30	25.39	16
Periplasmic protein CpxP *(cpxP)*	-0.21	2.15	28.88	28.81	28.63	28.60	28.56	28.53	30.57	30.62	30.92	38

[a] DiGiuseppe PA, Silhavy TJ. Signal detection and target gene induction by the CpxRA two-component system. J Bacteriol 2003;185:2432–40.

[b] Kumari S, Beatty CM, Browning DF, Busby SJW, Simel EJ, Hovel-Miner G. Regulation of acetyl coenzyme A synthetase in escherichia coli. J Bacteriol 2000;182:4173–9.

[c] Raivio TL, Popkin DL, Silhavy TJ. The Cpx envelope stress response is controlled by amplification and feedback inhibition. J Bacteriol 1999;181:5263–72.

[d] Venkova T, Wegrzyn G, Brom S, Nesvera J, Srivastava P, Jaishankar J. Molecular Basis of Stationary Phase Survival and Applications. Front Microbiol 2017;8.
