# Supplementary material for: Yersinia actively downregulates type III secretion and adhesion at higher cell densities
Source: PLoS Pathog. 2025 Aug 12;21(8):e1013423. doi: 10.1371/journal.ppat.1013423 (PMC12404644; doi:10.1371/journal.ppat.1013423)
Supplement: S4 Table — Label-free quantitative mass spectrometry quantification of CsrA in the total proteome of a ΔHOPEMTasd wild-type strain at the different growth conditions indicated, experiment and display format as shown in Table 1. (PDF) [file ppat.1013423.s018.pdf]

S4 Table – CsrA levels are not not significantly altered at different densities.

Label-free quantitative mass spectrometry quantification of CsrA in the total proteome of a ΔHOPEMTasd wild-type strain at the different growth conditions indicated, experiment and display format as shown in Table 1.

Protein	Log_2 intensity ratio	*p* value	Individual replicate log_2 intensity values						# pept.
			OD_{in} = 0.1			OD_{in} = 1.5			
CsrA	0.52	0.17	23.82	24.56	25.06	24.83	25.01	25.15	7
