# Supplementary material for: Yersinia actively downregulates type III secretion and adhesion at higher cell densities
Source: PLoS Pathog. 2025 Aug 12;21(8):e1013423. doi: 10.1371/journal.ppat.1013423 (PMC12404644; doi:10.1371/journal.ppat.1013423)
Supplement: S5 Table — Expression of genes affected by the absence of CsrA in Y. pseudotuberculosis [e]. All genes with gene identifier and with a > 4-fold transcription change in the csrA mutant were obtained from the data in the “Yersiniomics database” [f ] (https://yersiniomics.pasteur.fr). Flagellar genes, which are not expressed at 37°, were excluded. Label-free quantitative mass spectrometry in the total proteome of a ΔHOPEMTasd wild-type strain at the different growth conditions indicated, experiment and display format as shown in Table 1. (PDF) [file ppat.1013423.s019.pdf]

S5 Table – Density-dependent regulation of expression of proteins with CsrA-dependent transcription in *Y. pseudotuberculosis*.

Expression of genes affected by the absence of CsrA in *Y. pseudotuberculosis* at 25°C [e]. All genes with gene identifier and with a >4-fold transcription change in the *csrA* mutant were obtained from the data in the "Yersiniomics database" [f] (https://yersiniomics.pasteur.fr). Flagellar genes, which are not expressed at 37°, were excluded. Label-free quantitative mass spectrometry in the total proteome of a ΔHOPEMTasd wild-type strain at the different growth conditions indicated, experiment and display format as shown in Table 1.

Protein	Log_2 intensity ratio	*p* value	Individual replicate log_2 intensity values						# pept.
			OD_{in} = 0.1			OD_{in} = 1.5			
Proteins upregulated in *Y. pseudotuberculosis* ΔcsrA									
Urease accessory protein UreE	3.23	8.54E-05	27.67	28.63	28.69	31.69	31.61	31.39	41
Pyruvate dehydrogenase PoxB	2.60	5.38E-04	18.85	19.90	20.00	21.98	22.09	22.47	5
Glycerol kinase GlpK	1.83	2.88E-07	27.86	27.97	28.02	29.80	29.73	29.81	27
Osmotically-inducible protein Y, OsmY	1.29	1.09E-04	28.61	28.82	28.92	29.87	30.15	30.21	13
T6SS Protein Hcp1	1.03	1.78E-03	23.82	24.19	23.82	24.69	25.00	25.24	14
Periplasmic serine endoprotease DegP	0.74	1.12E-04	31.05	31.01	31.00	31.91	31.70	31.68	47
Protein TolB	0.63	1.67E-05	28.72	28.75	28.78	29.42	29.40	29.33	23
Imidazole glycerol phosphate synthase subunit HisF	0.44	5.58E-05	26.29	26.27	26.28	26.74	26.74	26.68	14
UTP--glucose-1-phosphate uridylyltransferase GalU	0.27	1.84E-03	29.40	29.50	29.44	29.76	29.66	29.73	30
Transcriptional repressor MprA	0.14	2.44E-02	25.95	25.90	25.96	26.05	26.04	26.13	9
Imidazoleglycerol-phosphate dehydratase HisB	0.06	1.56E-01	25.59	25.57	25.60	25.65	25.63	25.64	13
Histidinol dehydrogenase HisD	-0.23	1.29E-03	26.63	26.57	26.62	26.35	26.39	26.38	10
Histidinol-phosphate aminotransferase HisC	-0.51	1.08E-04	25.70	25.69	25.67	25.09	25.22	25.23	7
Proteins downregulated in *Y. pseudotuberculosis* ΔcsrA									
Cold shock-like protein CspE	0.80	5.56E-06	26.78	26.79	26.85	27.56	27.65	27.61	3
Acyl carrier protein AcpP	-0.16	6.78E-03	30.47	30.44	30.41	30.31	30.28	30.25	10
Aspartokinase LysC	-0.49	7.80E-04	26.86	26.82	26.79	26.25	26.26	26.47	21
RNA-binding protein YhbY	-0.64	2.37E-05	27.77	27.77	27.74	27.08	27.20	27.08	1
50S ribosomal protein L31, RpmE	-0.68	3.92E-05	30.20	30.10	30.12	29.50	29.49	29.37	6
30S ribosomal protein S20, RpsT	-0.96	5.70E-05	30.99	30.97	30.99	30.01	30.18	29.89	14

[e] Bücker R, Heroven AK, Becker J, Dersch P & Wittmann C (2014) The pyruvate-tricarboxylic acid cycle node: A focal point of virulence control in the enteric pathogen Yersinia pseudotuberculosis. J. Biol. Chem. 289: 30114–30132

[f] Lê-Bury P, Druart K, Savin C, Lechat P, Mas Fiol G, Matondo M, Bécavin C, Dussurget O & Pizarro-Cerdá J (2023) Yersiniomics, a Multi-Omics Interactive Database for Yersinia Species. Microbiol. Spectr. 11: e0382622
