# Supplementary material for: Yersinia actively downregulates type III secretion and adhesion at higher cell densities
Source: PLoS Pathog. 2025 Aug 12;21(8):e1013423. doi: 10.1371/journal.ppat.1013423 (PMC12404644; doi:10.1371/journal.ppat.1013423)
Supplement: S6 Table — (PDF) [file ppat.1013423.s020.pdf]

S6 Table – Strains and plasmids used in this study

Strain	Genotype	Reference
MRS40	Wild-type *Y. enterocolitica* E40 *ΔblaA*	[g]
IML421*asd* (ΔHOPEMTasd)	MRS40 *yopH*$_{\Delta 1\text{-}352}$ *yopO*$_{\Delta 65\text{-}558}$ *yopP*$_{43}$ *yopE*$_{5}$ *yopM*$_{18}$ *yopT*$_{135}$ *Δasd*	[h]
AD4085	IML421*asd egfp-sctQ*	[h]
AD4615	MRS40 P$_{yopE}$::*sfGFP-ssrA*	This study
CH4010	MRS40 *mCherry-sctL sctG-sfgfp*	[i]
FE005	IML421*asd* P$_{yopE}$::*sfGFP-SsrA ΔyadA*	This study
FE007	IML421*asd* P$_{yopE}$::*sfGFP-SsrA ΔsctW*	This study
FE014	IML421*asd ΔvirF*	[j]
FE015	MRS40 *ΔrpoS*	This study
FE022	IML421*asd ΔyenI ΔlsrK*	This study
FE027	P$_{yopE}$::*sfGFP-SsrA ΔcsrC*	This study
FE028	P$_{yopE}$::*sfGFP-SsrA ΔrelA*	This study
FE029	P$_{yopE}$::*sfGFP-SsrA ΔspoT*	This study

Plasmids	Genotype	Reference
pAD477	pBAD::mCherry<br>(expression plasmid for mCherry expression)	[k]
pAD492	pBAD::EGFP<br>(expression plasmid for EGFP expression)	[i]
pAD716	pKNG101-PyopE-sfGFP-SsrA<br>(mutator for P*yopE* reporter system)	This study
pBAD-His B	pBR322-derived expression vector	Invitrogen
pFE002	pKNG101-*ΔrpoS*<br>(mutator for deletion of the *rpoS* gene)	This study
pFE003	pBAD::*rpoS*<br>(expression plasmid for RpoS expression)	This study
pFE010	pBAD::*virF*<br>(expression plasmid for VirF expression)	[j]
pFE018	pKNG101-*ΔyenI*<br>(mutator for deletion of the *yenI* gene)	This study
pFE021	pKNG101-*ΔlsrK*<br>(mutator for deletion of the *lsrK* gene)	This study
pFE022	pBAD::*csrA*<br>(expression plasmid for CsrA overexpression)	This study
pFE025	pKNG101-*ΔcsrC*<br>(mutator for disruption of the *csrC* gene)	This study
pFE026	pKNG101-*ΔrelA*<br>(mutator for disruption of the *relA* gene)	This study
pFE027	pKNG101-*ΔspoT*<br>(mutator for disruption of the *spoT* gene)	This study
pLJM31	pKNG101-*ΔyadA*<br>(mutator for disruption of the *yadA* gene)	[l]
pKNG101	*oriR6K sacBR*$^{+}$ *oriTRK2 strAB*$^{+}$<br>(suicide vector for homologous recombination)	[m]

[g] Sory M-P, Boland A, Lambermont I, Cornelis GR. Identification of the YopE and YopH domains required for secretion and internalization into the cytosol of macrophages, using the cyaA gene fusion approach. Proc Natl Acad Sci U S A 1995;92:11998–2002.

[h] Kudryashev M, Stenta M, Schmelz S, Amstutz M, Wiesand U, Castaño-Díez D, et al. In situ structural analysis of the Yersinia enterocolitica injectisome. Elife 2013;2:e00792.

[i] Wimmi S, Fleck M, Helbig C, Brianceau C, Langenfeld K, Szymanski WG, et al. Pilotins are mobile T3SS components involved in assembly and substrate specificity of the bacterial type III secretion system. Mol Microbiol 2024;2:304–23.

[j] Schott S, Scheuer R, Ermoli F, Glatter T, Evguenieva-Hackenberg E, Diepold A, et al. A ParDE toxin-antitoxin system is responsible for the maintenance of the Yersinia virulence plasmid but not for type III secretion-associated growth inhibition. Front Cell Infect Microbiol 2023;13:1166077.

[k] Diepold A, Sezgin E, Huseyin M, Mortimer T, Eggeling C, Armitage JP. A dynamic and adaptive network of cytosolic interactions governs protein export by the T3SS injectisome. Nat Commun 2017;8.

[l] Kudryashev M, Stenta M, Schmelz S, Amstutz M, Wiesand U, Castaño-Díez D, et al. In situ structural analysis of the Yersinia enterocolitica injectisome. Elife 2013;2013.

[m] Kaniga K, Delor I, Cornelis GR. A wide-host-range suicide vector for improving reverse genetics in Gram-negative bacteria: inactivation of the blaA gene of Yersinia enterocolitica. Gene 1991;109:137–41.
