# Supplementary material for: Yersinia actively downregulates type III secretion and adhesion at higher cell densities
Source: PLoS Pathog. 2025 Aug 12;21(8):e1013423. doi: 10.1371/journal.ppat.1013423 (PMC12404644; doi:10.1371/journal.ppat.1013423)
Supplement: S7 Table — Nucleotide sequence of oligonucleotides used for the construction of plasmids or quantitative PCR (qPCR). (PDF) [file ppat.1013423.s021.pdf]

**S7 Table – Oligonucleotides used in this study**

Nucleotide sequence of oligonucleotides used for the construction of plasmids or quantitative PCR (qPCR).

| Primer name | Sequence (5´→ 3´) | Used for |
|---|---|---|
| AD1117 | AAGGTCTCGGGCCCCGATAACCGGTTCAATAGTATCTGG | pAD716 |
| AD1118 | GTCCTTGTAGTCACCTCCCAATTGAAAGATCTTATTTTCATGACTATTTATTCCCTTGGCT | pAD716 |
| AD1119 | CAATTGGGAGGTGACTACAAGGACGACGATGATAAGTGATATGGATAAAAACAAGGGGGTAG | pAD716 |
| AD1120 | AAGGTCTCTCTAGAGATTTGCTCTGACATGCGCC | pAD716 |
| AD1498 | TATAGGTCTCGGGCCCTGAATCGAAAGTAACTTGCGGTTGGTAG | pFE002 |
| AD1499 | TATAGGTCTCTCTAGATTACTCGGTTCATGATCTAATTTATGA | pFE002 |
| AD1500 | TATACCATGGGTAGCCAAAATACGCTGAAAGTTAACGAGT | pFE003 |
| AD1501 | TATAAAGCTTTTCGCGGAACAATGCTTCGATGCTCAGG | pFE003 |
| AD1835 | TATAAAGCTTGCCTGTGGTTGCTATTTTAGTAAGAC | pFE010 |
| AD1856 | TATAGGTCTCCCATGGGTGCATCACTAGAGATTATTAAATTAGAATGGGC | pFE010 |
| AD1901 | TATAGGTCTCGGGCCCTATCTCCAATGGAAGCGACGATAGTATC | pFE018 |
| AD1902 | TTTTTATTTTAGCTTCAACTCAATGCCAAGC | pFE018 |
| AD1903 | GAAGCTAAAATAAAAACCAAAGTTATTTAATTAAAACAACATCGT | pFE018 |
| AD1904 | AAGGTCTCTCTAGAGACGAGAAAAACAAATTTTTTATTAGTAAAAAGTGG | pFE018 |
| AD1940 | TATAGGTCTCGGGCCCAGGATGCGCCATCATCAGCAACG | pFE021 |
| AD1941 | GATCGAACAAAAATTAGCTATCTTACTGGCCAGCT | pFE021 |
| AD1942 | GCTAATTTTTGTTCGATCCTCAGCTTCAATGTG | pFE021 |
| AD1943 | AAGGTCTCTCTAGACTGATGAGTTGACCGCAAGATCA | pFE021 |
| AD2232 | TATAGGTCTCGGGCCCCTGAAGGTGACTCACCCAG | pFE025 |
| AD2233 | TATAGGTCTCTCTAGAGATGTACTGGCAAGGAGCG | pFE025 |
| AD2234 | TATAGGTCTCGGGCCCACATATACCCGATCGTCAAATACC | pFE026 |
| AD2235 | TATAGGTCTCTCTAGAGCAGATGAAGGGATCAAAGCC | pFE026 |
| AD2236 | TATAGGTCTCGGGCCCTAAAGAAGTCGATACGTGCTATCG | pFE027 |
| AD2237 | TATAGGTCTCTCTAGAAAAGTGGATAGGGCTGGCG | pFE027 |
| AD2032 | TATACCATGGGTCTTATTCTGACTCGTCGAGTTGGT | pFE022 |
| AD2033 | TATAAAGCTTCAGTAAGTCGTCGGTTGAGACT | pFE022 |
| AD1977 | TCACCAACAACATTCCACAG | qPCR *gyrB* |
| AD1978 | TTCGACCGCAGTTTTTACC | qPCR *gyrB* |
| AD1981 | CCATTATCTCGCACAGCAC | qPCR *sctG* |
| AD1982 | TAAAGTTCACCTTCCCCGC | qPCR *sctG* |
| AD1983 | AGACGAGACAATGCCACAC | qPCR *virF* |
| AD1984 | GCAGAGCCGAGAGGAATAAAG | qPCR *virF* |
| AD2037 | GACGGTATCAGGATGGTGCC | qPCR *csrB* |
| AD2038 | TCCATCCTGGAGGTGTCCTT | qPCR *csrB* |
| AD2039 | TCCGGCCTGTGTCCATAAAC | qPCR *csrC* |
| AD2040 | AACAGTGCGGGATGTACTGG | qPCR *csrC* |
