# Supplementary material for: Yersinia actively downregulates type III secretion and adhesion at higher cell densities
Source: PLoS Pathog. 2025 Aug 12;21(8):e1013423. doi: 10.1371/journal.ppat.1013423 (PMC12404644; doi:10.1371/journal.ppat.1013423)
Supplement: S1 File — (PDF) [file ppat.1013423.s022.pdf]

Foci quantification with StarDist and ThunderSTORM

This manual describes the procedure to identify and count fluorescent foci in bacterial cells using deep-learning (DL) based segmentation and spot fitting. Segmentation is performed using StarDist and foci localization using ThunderSTORM, a popular Fiji plugin for single-molecule imaging.

In this protocol, foci of fluorescently labeled T3SS in *Yersinia enterocolitica* should be counted in 3D image stacks containing DIC and fluorescent images. However, the described manual can also be used in different setups with modifications.

Protocol:

Step 1: Cell segmentation using StarDist

StarDist is a deep-learning based segmentation algorithm developed by Uwe Schmidt in the group of Gene Myers (MPI-CBG, Dresden). You can find details on the Github repository or in the publication.

It works well for small and oval/round bacteria as shown in the DeepBacs project (Github wiki and Spahn et al., 2022), but can also be applied to *Yersinia*, when manually curating the detected cells (Fig. 1).

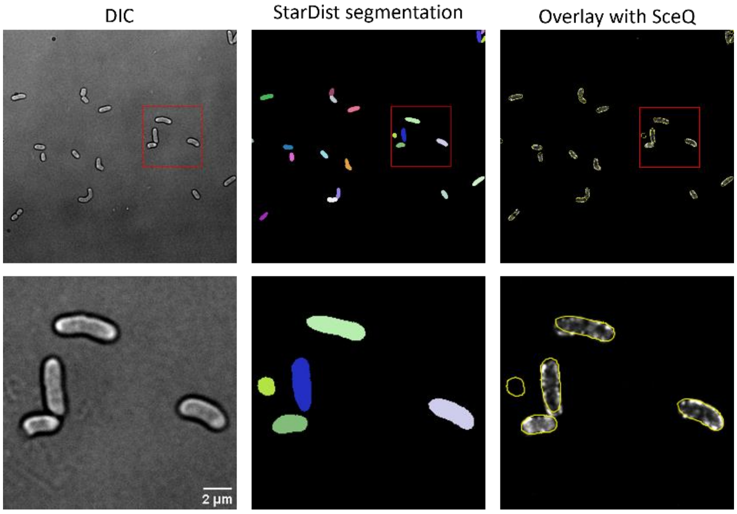

Figure 1: Example for segmentation of Yersinia enterocolitica DIC images using StarDist.

To train our segmentation model, we use the StarDist notebook that is implemented in the ZeroCostDL4Mic project (Github wiki and von Chamier et al., 2021). It uses Google Colaboratory and requires a Google account and data uploaded to the Google Drive. Documentation on StarDist and guidelines can be found in the StarDist, ZeroCostDL4Mic and DeepBacs publications. In brief, StarDist is based on U-Net (Fig. 2), which learns to map an input (bright light image) to an output (segmented image).

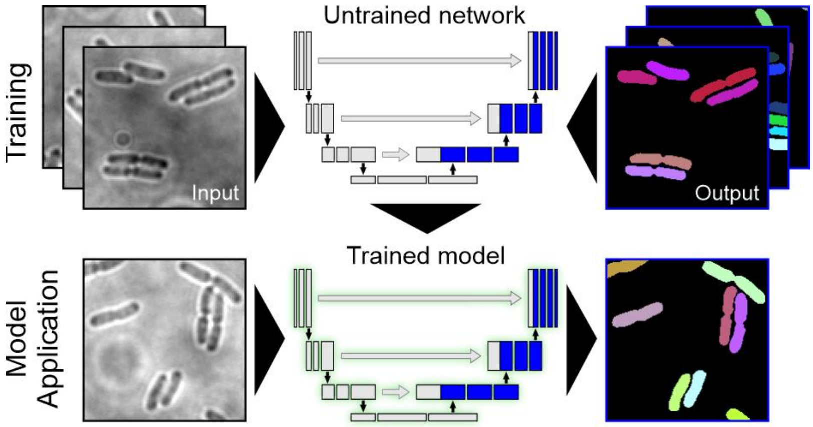

Figure 2: Schematic of DL-based segmentation (Source: DeepBacs wiki)

Once a model is trained online, it can be downloaded and used directly in Fiji.

To use this workflow, **StarDist has to be installed**, which can be conveniently done via the Update sites ("Help" -> "Update…" -> Press on "Manage Update site" button and tick "StarDist"). **ThunderSTORM can be installed using the "Hohlbein Lab" update site**.

<u>The sequence for segmenting is the following:</u>

- Open the image stack in Fiji and duplicate the slice in which bacteria can be segmented most reliable (depends on the trained model).
- Run "StarDist 2D" by selecting "Model (zip) from File" and adding the parameters that are specific to the model (can be found in the "Threshold.json" file of the StarDist model)
- Curate the ROIs, i.e. remove false positives (dirt or background), incompletely segmented or strongly overlapping cells by clicking on the ROI number in the image and pressing Delete ("Show All with Labels has to be activated in ROI manager)
- Once the ROIs are curated, save them in the analysis folder

Step 2: Identify foci using ThunderSTORM

Execute ThunderSTORM via the command “Run Analysis”, which can be found in the menu (Plugins -> ThunderSTORM).

ThunderSTORM uses Wavelet analysis to identify local maxima and fits these with a function of choice. In our case, we will use a 2D Gaussian fit function.

Start by duplicating the fluorescent channel/slice to be processed. Afterwards, open ThunderSTORM using “Run Analysis”. A window with options and parameters will open (see figure below).

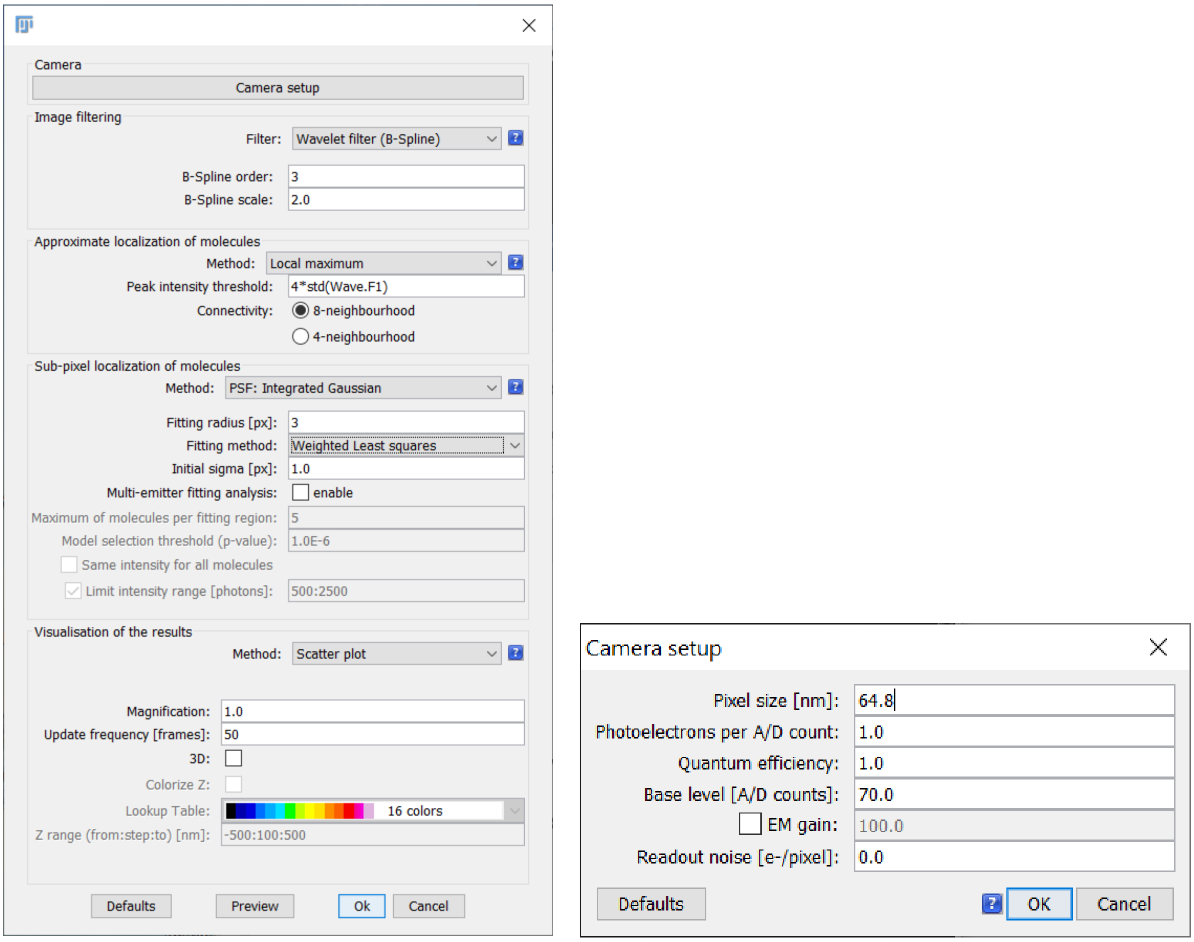

Figure 3: ThunderSTORM plugin appearance and the Camera setup sub-menu.

In order to provide properly scaled results, the pixel size has to be added in the “**Camera setup**” menu. The base level can be added as an additional parameter for the fitting algorithm. For this, select a background region and extract the mean value using the “Histogram” function. A/D count is not important here, as we do not require the localization precision like in super-resolution microscopy.

As we want to identify foci based on their surroundings, “**Local Maximum**” is chosen as a method. The peak intensity is set to a certain multiple of the standard deviation in the first-order Wavelet image, which can be interpreted as a certain Signal-to-Noise ratio. Higher multiples will only give you brighter spots, while lower multiple give you also dim spots (but also more false positives). Check a suitable multiple with proper controls.

The “**Fitting radius**” depends on the size of the clusters (and thus on the pixel size). For the pixel size here (~65 nm), 3 px give good results, but it can be tweaked if localization is not satisfying. *Too large windows will lead to shifted localizations.*

The “**Initial sigma [px]**” also depends on your foci size and should be chosen in the range of 1-3 px. It does not change the results significantly, but a proper parameter can speed up analysis in super-resolution imaging, where you have thousands of frames. Here, 1 or 2 px are fine.

Visualization:

Visualization is key, as we want to count the number of identified foci. In super-resolution microscopy, we require a magnified image due to the higher resolution, but in this protocol, we need an output image of the same size as our input image. **The Magnification thus has to be set to 1**. Update frequency and 3D is not of interest here.

Once set all parameters, press “**Preview**” to check whether they provide reasonable results. Several images will open (Wavelet image, log file with threshold, example detection window). The image with the detections will update when you change parameters and press “Preview” again.

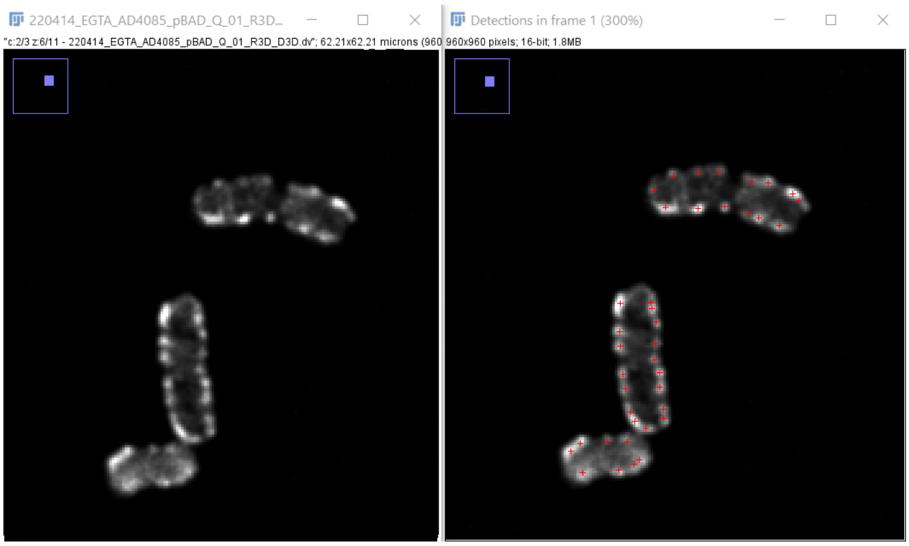

Figure 4: Example of the Preview image.

If satisfied with the result, close the preview image and run ThunderSTORM analysis by pressing “Ok” at the very bottom of the plugin window. A table with the localizations will open together with an overlay image of the detected foci (like above) and the scatter plot image (see below). The **localization list** provides information about each focus that was found in the image (coordinates, intensity, sigma aka standard deviation of the Gaussian fit).

This list can be filtered to remove false localizations:

Filtering parameters:

sigma < 600 (can be varied based on the spot size)

- ➔ This filters out localizations from asymmetric peaks or peaks that are out of focus
- ➔ Applies to most peaks that were detected in the continuous membrane
- ➔ Can be reduced to get rid of more elongated signals

Type in the command in the “Filter” field (here: sigma < 600) and press “**Apply**”. Filters can also be combined, but this is not important here.

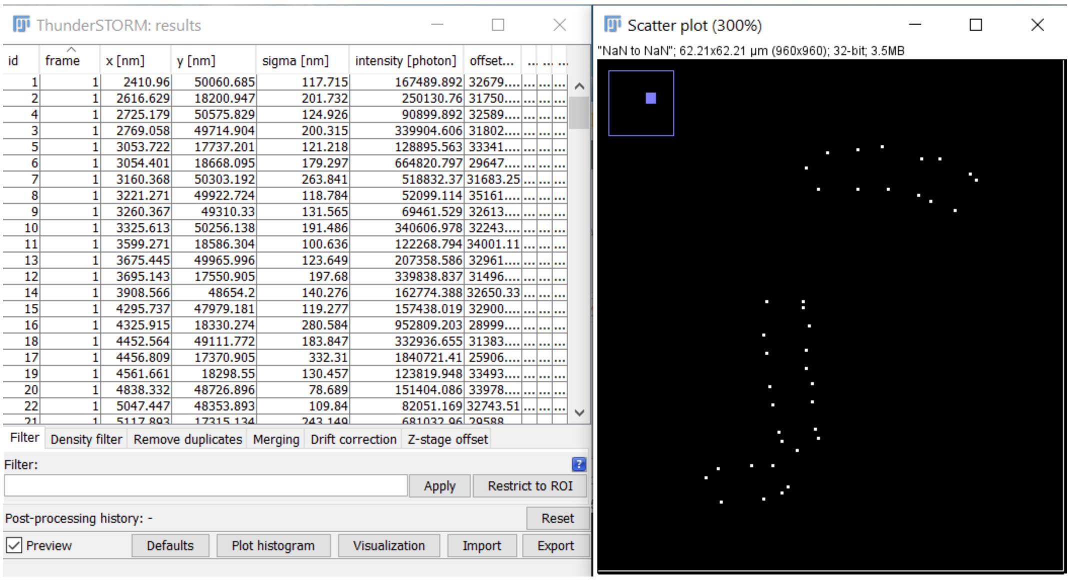

Figure 5: Localization list and Scatter plot image.

If satisfied with the result, export the localization list ("Export") and save the Scatter plot image.

In the Scatter Plot image, each localized focus adds one gray value to the centroid pixel, making it easy to quantify the number of foci by determining the intensity in each cell.

Step 3: Counting the localization in the Scatter plot image

To count the number of foci per cells, the segmented ROIs (likely still open in the ROI manager) and the Scatter plot image are needed. As the foci are located on the membrane, some localizations might not be inside the ROIs that were generated using StarDist.

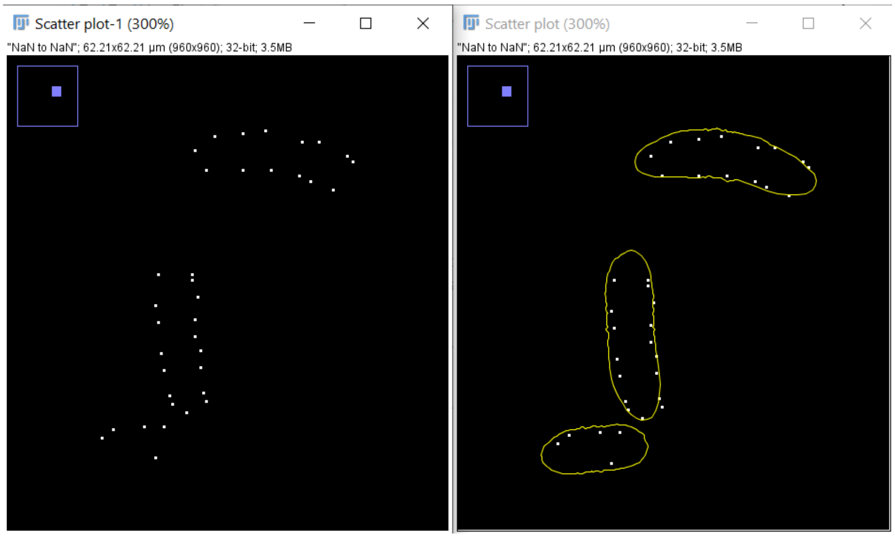

Figure 6: Overlay of foci Scatter plot and StarDist segmentation

To correct for this, we use a custom macro that enlarges each ROI by a specific number of pixels and measures the intensity for each cell. This intensity corresponds to the number of foci per cell.

To run the macro, open the macro window ("Plugins" -> "New" -> "Macro"; set language to IJ1 Macro) and paste the code provided at the end of the document. Alternatively, the macro can be saved and

opened from the Macro window. The “**enlarge_diam**” can be adjusted to make the ROIs even bigger or smaller (negative numbers will shrink the ROI).

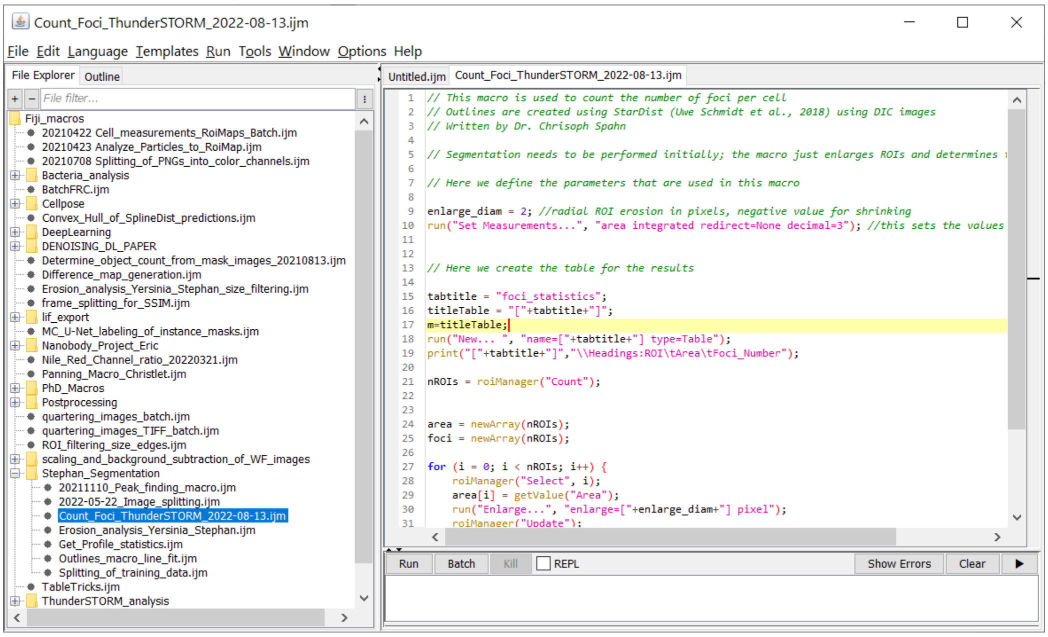

Figure 7: Fiji macro window.

To execute the macro, press “Run”. This should adjust the ROI sizes, measure the intensity and create a results table with the number of detected spots per cell, as well as the cell area. You can save the table (and eventually also the adjusted ROIs) and use it for further processing.

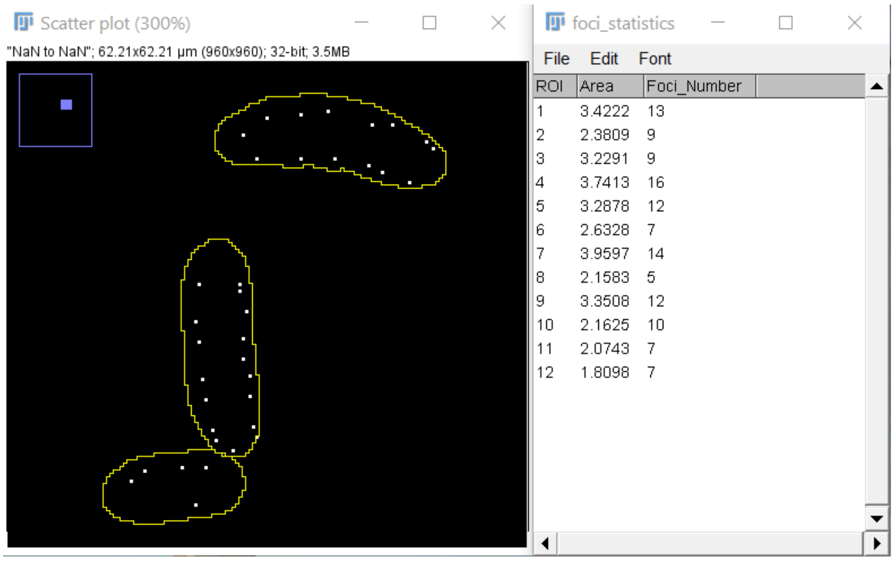

Figure 8: Results of the foci quantification macro.

Macro code for ROI enlargement and foci counting

```
// This macro is used to count the number of foci per cell
// Outlines are created using StarDist (Uwe Schmidt et al., 2018) using DIC images
// Written by Dr. Christoph Spahn

// Segmentation needs to be performed initially; the macro just enlarges ROIs and determines the molecule number

// Here we define the parameters that are used in this macro

enlarge_diam = 2; //radial ROI erosion in pixels, negative value for shrinking
run("Set Measurements...", "area integrated redirect=None decimal=3"); //this sets the values that are measured


// Here we create the table for the results

tabtitle = "foci_statistics";
titleTable = "["+tabtitle+"]";
m=titleTable;
run("New... ", "name=["+tabtitle+"] type=Table");
print("["+tabtitle+"]","\\Headings:ROI\tArea\tFoci_Number");

nROIs = roiManager("Count");


area = newArray(nROIs);
foci = newArray(nROIs);

for (i = 0; i < nROIs; i++) {
		roiManager("Select", i);
		area[i] = getValue("Area");
		run("Enlarge...", "enlarge=["+enlarge_diam+"] pixel");
		roiManager("Update");
		roiNumber = i+1;
		foci[i] = getValue("RawIntDen");
		print("["+tabtitle+"]", roiNumber + "\t" +area[i] +"\t"+foci[i]);
}
```
