# Supplementary material for: Yersinia actively downregulates type III secretion and adhesion at higher cell densities
Source: PLoS Pathog. 2025 Aug 12;21(8):e1013423. doi: 10.1371/journal.ppat.1013423 (PMC12404644; doi:10.1371/journal.ppat.1013423)
Supplement: Attachment — Submitted filename: Response to Reviewers.docx [file ppat.1013423.s025.docx]

Dear Dr. Diepold,

Thank you for submitting your manuscript to PLOS Pathogens. After careful consideration, we feel that it has merit but does not fully meet PLOS Pathogens's publication criteria as it currently stands. Therefore, we invite you to submit a revised version of the manuscript that addresses the points raised during the review process.

In particular, a reviewer remains hesitant to accept the model put forth. Either the experiments proposed by the reviewer should be completed and re-submitted or the model should be revised. Overall, we think the data are worth publishing and that they meet the rigorous standards of PLOS Pathogens. However, we cannot accept the manuscript as currently written.

Please submit your revised manuscript within 60 days Aug 31 2025 11:59PM. If you will need more time than this to complete your revisions, please reply to this message or contact the journal office at plospathogens@plos.org. When you're ready to submit your revision, log on to https://www.editorialmanager.com/ppathogens/ and select the 'Submissions Needing Revision' folder to locate your manuscript file.

Please include the following items when submitting your revised manuscript:

* A rebuttal letter that responds to each point raised by the editor and reviewer(s). You should upload this letter as a separate file labeled 'Response to Reviewers'. This file does not need to include responses to any formatting updates and technical items listed in the 'Journal Requirements' section below.

* A marked-up copy of your manuscript that highlights changes made to the original version. You should upload this as a separate file labeled 'Revised Manuscript with Track Changes'.

* An unmarked version of your revised paper without tracked changes. You should upload this as a separate file labeled 'Manuscript'.

If you would like to make changes to your financial disclosure, competing interests statement, or data availability statement, please make these updates within the submission form at the time of resubmission. Guidelines for resubmitting your figure files are available below the reviewer comments at the end of this letter.

We look forward to receiving your revised manuscript.

Kind regards,

Jon Paczkowski
Academic Editor
PLOS Pathogens

David Skurnik
Section Editor
PLOS Pathogens

Sumita Bhaduri-McIntosh
Editor-in-Chief
PLOS Pathogens

Michael Malim
Editor-in-Chief
PLOS Pathogens

Dear members of the editorial team,

We thank the reviewer for the renewed input und you for your assessment and the clear instructions.

To briefly recapitulate, the critique of reviewer #2 is on a specific part of the proposed regulatory pathway, namely on the connection marked by asterisks in the following representation of events leading to the effect described in our manuscript, the VirF-mediated downregulation of the T3S at high densities:

High bacterial densities -> higher concentrations of regulatory RNAs csrB and csrC -> sequestration of CsrA [->*] [CsrA can no longer bind to and stabilize *virF* mRNA*] -> decreased levels of VirF -> decreased levels of T3SS structural components and adhesin YadA -> decreased T3SS assembly and adhesion -> switch from colonization to replication and dissemination state.

In the manuscript, we directly show most of these points and connections, including that at high cell densities, the level of the regulatory RNAs csrB and, more strongly, csrC are increased, and all points from the decreased levels of VirF on.

The part of the scheme we did not directly test was the connection between the increased csrB/C levels and virF expression, as these connections were already published in other circumstances by highly respected researchers: The sequestration of CsrA by CsrB/C was shown in a number of publications (reviewed in Heroven *et al.*, RNA Biol (2012), Kusmierek *et al*., Curr Opin Microbiol (2018), Volk *et al*., Curr Top Microbiol Immunol (2020)), and the group of Petra Dersch showed in in a paper in PLOS Pathogens that CsrA in *Y. pseudotuberculosis* directly binds to the *virF*/*lcrF* transcript and stabilizes it, which leads to an increased expression of VirF (Kusmierek *et al*., PLOS Pathogens 2019). We had not re-tested this link in the original version of the manuscript, because the *virF* sequence is 98% identical in *Y. enterocolitica* (now shown in Suppl. Fig. 13). Nevertheless, as requested by the reviewer in the previous round of revision, we performed a VirF reporter experiment using the CsrA binding mutant described in the Kusmierek *et al*. paper. As explained in more detail in the response to the reviewer, expression of the VirF reporter was decreased both at high densities and in the CsrA binding mutant of the reporter (as expected), but was also further decreased in the high density CsrA binding mutant control additionally requested by the reviewer. This is likely due to the fact that (i) the binding site mutant does not fully inhibit CsrA binding, but reduces it by a factor of 2-4, as shown in Fig. 2B of Kusmierek *et al.* (2019), and that (ii) the mutation, which is in the ribosome binding site, may reduce ribosome binding as well as CsrA binding. We concede that these results, while in line with a direct link between CsrA and VirF levels, do not prove it, and have therefore removed the respective Supplementary Figure. Nevertheless, the increase of the regulatory RNA csrC at high densities and the high similarity between the *Y. pseudotuberculosis* and *Y. enterocolitica* *virF* sequence provide strong evidence that this pathway, (rather than an unknown pathway excluding all candidates tested negative in our manuscript and any pathway involving significant protein level changes, which we would have detected in the proteomic results), is responsible for the connection between CsrC increase and VirF decrease at high bacterial densities.

As suggested by the editors, we have accordingly revised our description of the model to clarify that the direct link between CsrA and VirF, while shown by the previous PLOS Pathogens paper of Kusmierek and colleagues for the *Y. pseudotuberculosis* system and in good agreement with our data, was not shown for *Y. enterocolitica* in our manuscript. To this aim, we made a large number of changes in the manuscript, in order to specifically describe where findings and resulting conclusions are based on data shown in the manuscript, and where our reasoning is based on findings from the *Y. pseudotuberculosis* system (namely, for the connection between CsrA and VirF levels, where we also list the respective reference(s)). The exact changes are listed in the reply to the reviewer below.

We think that this revision of the description of the model, as suggested by the editors, and the clearer specification of the data underlying the individual findings and conclusions, has further increased the rigor of the manuscript and hope that this revised version meets the requirements for publication in PLOS Pathogens.

Reviewers' Comments:

Reviewer's Responses to Questions

Part I - Summary

Please use this section to discuss strengths/weaknesses of study, novelty/significance, general execution and scholarship.

Reviewer #2: My main concern with the original submission was the lack of compelling evidence to support the idea that the effect of cell density on T3SS gene expression was due to CsrA. The authors have made two changes to the manuscript to address this question. First, they show that protein levels for other CsrA-regulated genes are affected much less by cell density than protein levels for T3SS components. They argue that the effects of CsrA are lower when the T3SS is expressed, but the effects of CsrA on the T3SS would presumably also be lower if that were the case. Second, the authors made a virF translational reporter construct, and looked at the effect of mutating the CsrA binding site. Their data support the idea that CsrA regulates virF expression. If their model is correct, you would expect that the expression of the mutant reporter fusion would be unaffected by cell density, but they omit this key experiment. Moreover, the relatively small effect of mutating the CsrA binding site compared to the effect of cell density argues against a role for CsrA in the cell density-dependent regulation. Similarly, deleting csrC has little impact on density-dependent regulation, although the authors argue that autoregulation in the Csr system may buffer the effect of deleting csrC. Overall, I remain unconvinced of the role of CsrA in the density-dependent regulation.

We thank the reviewer for the detailed statement. To recapitulate, in the manuscript, we directly show that at high cell densities, the level of the regulatory RNAs csrB and, more strongly, csrC are increased. We then again directly show the decreased levels of virF and the resulting decreased expression, assembly and function of the T3SS, and the concurrent downregulation of the adhesion YadA, which together lead to the described phenotype. The critique of the reviewer focuses on the proposed link between the increased csrB/C levels and the virF levels via CsrA. Indeed, we did not directly test this link in our initial manuscript, as it has been shown by respected research groups in different circumstances: It was shown in several publications that CsrB and CsrC sequester CsrA (reviewed in Heroven *et al.*, RNA Biol (2012), Kusmierek *et al*., Curr Opin Microbiol (2018), Volk *et al*., Curr Top Microbiol Immunol (2020)). The group of Petra Dersch found that CsrA directly binds to the *virF*/*lcrF* transcript and stabilizes it, which leads to an increased expression of VirF (Kusmierek *et al*., PLOS Pathogens (2019)). The *virF* upstream and coding sequence is 98% identical between *Y. pseudotuberculosis* and *Y. enterocolitica* (which we show in the new Suppl. Fig. 13), making a similar connection likely. Nevertheless, as requested by the reviewer in the previous comments, we performed a VirF reporter experiment using the CsrA binding mutant described in the Kusmierek *et al*. paper. As expected, expression of the VirF reporter was decreased both at high densities and in the CsrA binding mutant of the reporter; the VirF levels were slightly higher in the mutant than at high densities. The reviewer requested the double negative control, the CsrA binding mutant at high densities. This is indeed an important additional control: As the reviewer states, if the density-dependent downregulation of VirF is caused by CsrA and CsrA is completely sequestered by CsrB/C at high bacterial densities, one would expect no further reduction of VirF levels. We have now performed this experiment, both using the reporter construct used in the previous Suppl. Fig. 13 (which comprises the CsrA binding region defined by Kusmierek *et al*.), and a translational reporter comprising this region and the full-length virF gene, as originally proposed by the reviewer (we succeeded in cloning this construct – which might have a secondary effect due to the role of the VirF protein itself – after the submission of the previous revised version.) In both cases, the VirF levels of the CsrA binding mutant at ODin = 1.5 were indeed further reduced compared to the levels of the CsrA binding mutant at ODin = 0.1. This can be explained by the fact that (i) the binding site mutant does not fully inhibit CsrA binding – it is reduced it by a factor of 2-4, as shown in the Kusmierek *et al.* paper (Fig. 2B) – or that (ii) the mutation, which is in the ribosome binding site, may reduce ribosome binding as well as CsrA binding. We concede that, while these results are in line with a direct link between CsrA and VirF, they are no proof for it, and thank the reviewer for raising this point. As suggested by the editors, we have accordingly revised our description of the model to highlight that the direct link between CsrA and VirF, while shown by the previous PLOS Pathogens paper of Kusmierek and colleagues for the highly similar *Y. pseudotuberculosis* system, and suggested by the increase of the regulatory RNA csrC at high densities, was not shown for *Y. enterocolitica* in our manuscript. Specifically, we changed the following passages of the manuscript (line numbers refer to the revised manuscript version without tracked changes):

- Abstract: Removed the passage “Transcript analysis showed that this effect is mediated by increased levels of the regulatory RNAs CsrBC, which sequester the regulatory protein CsrA and destabilize the virF transcript.”
- Lines 79-82: Changed “A >10 times increased concentration of the regulatory RNA CsrC at higher bacterial densities leads to sequestration of the RNA-binding protein CsrA and downregulation of expression of the main T3SS transcription factor VirF. Accordingly, expression of VirF *in trans* overrides the effect.“ to “At higher bacterial densities, we found a >10 times increased concentration of the regulatory RNA CsrC, which sequesters the RNA-binding protein CsrA. In the closely related *Y. pseudotuberculosis* system, CsrA sequestration was found to decrease the expression of the main T3SS transcription factor VirF (Kusmierek *et al*., 2019). Accordingly, we found that expression of VirF *in trans* overrides the effect.”
- Line 236: Changed the subheading “Bacterial density influences VirF levels at a post-transcriptional level via the CsrABC pathway” to “Increased concentrations of the post-transcriptional regulators CsrB and CsrC at higher bacterial density”
- Line 249-250: Changed “In *Yersinia*, CsrA modulates T3SS activation via *virF* transcript regulation.” to “In *Y. pseudotuberculosis*, CsrA modulates T3SS activation via *virF* transcript regulation (Kusmierek *et al*., 2019).”
- Lines 253-254: Inserted “The *virF* upstream and coding region are 98% identical between *Y. enterocolitica* and *Y. pseudotuberculosis* (Suppl. Fig. 13).”
- Lines 258-261: Changed “As CsrA levels remain relatively constant at the different bacterial densities (Suppl. Table 4), this suggests that the subsequent increased sequestration of CsrA by CsrC underlies the *virF* downregulation that causes d^3^T3 at higher bacterial densities. To more directly test an influence of CsrA on VirF expression, we constructed a reporter including the postulated CsrA binding region [55], *virF*(-123/+75)-sfGFP, expressed from plasmid. The resulting fluorescence after induction of the T3SS was decreased in the (-16/-14: GGA to TTC) mutant shown to reduce CsrA binding (Kusmierek *et al*., 2019) and at higher densities (Suppl. Fig. 13), in line with a direct link between CsrA and VirF levels.” to “As CsrA levels remain relatively constant at the different bacterial densities (Suppl. Table 4), this suggests a subsequent increased sequestration of CsrA by CsrC, which may underlie the *virF* downregulation that causes d^3^T3 at higher bacterial densities.”
- Lines 263-264: Changed “Bacterial density governs T3SS activity via posttranscriptional control of the *virF* transcript.” to “Increased abundance of the regulatory RNAs CsrBC at high bacterial density and posttranscriptional control of the *virF* transcript.”
- Lines 271-273: Changed “The regulatory RNAs CsrB and CsrC sequester CsrA, preventing it from binding and stabilizing the *virF* transcript.” to “The regulatory RNAs CsrB and CsrC sequester CsrA, which in the closely related *Y. pseudotuberculosis* system (Suppl. Fig. 13) prevents it from binding and stabilizing the *virF* transcript.”
- Lines 319-326: Changed “We found that the density-dependent downregulation of the T3SS is mediated by a pathway involving CsrABC and VirF, the main T3SS transcription factor in *Yersinia*. At higher bacterial densities, higher concentrations of the regulatory RNAs CsrB and CsrC sequester the regulatory protein CsrA, which usually stabilizes the *virF* transcript (Kusmierek *et al*., 2019; Meyer *et al.*, 2024) (Fig. 8b)*.* This leads to a gradual inhibition of the expression of VirF and, subsequently, all T3SS components, preventing the formation of injectisomes and protein secretion by the T3SS. The post-transcriptional nature of the effect of the CsrABC system on VirF (Meyer *et al.*, 2024) is illustrated by the finding...” to “Density-dependent downregulation of the T3SS may be mediated by a pathway involving CsrABC and VirF, the main T3SS transcription factor in *Yersinia*. At higher bacterial densities, higher concentrations of the regulatory RNAs CsrB and CsrC sequester the regulatory protein CsrA*.* In *Y. pseudotuberculosis*, CsrA was shown to bind to and most likely stabilize the *virF* mRNA, and sequestration of CsrA leads to a gradual inhibition of the expression of VirF (Kusmierek *et al*., 2019; Meyer *et al.*, 2024) (Fig. 8b). This reduction of VirF levels and, subsequently, all T3SS components at high *Y. enterocolitica* densities (Fig. 3a, Table 1, Suppl. Fig. 6), prevents the formation of injectisomes and protein secretion by
- Lines 335-337: Changed “While overall, we found that CsrABC gene regulation is comparable in our conditions (37°, T3SS-inducing conditions), the effect on the T3SS is stronger than on other genes affected by CsrABC (Fig. 3a, Suppl. Table 5).” to “While overall, we found that gene regulation is comparable in our conditions (37°, T3SS-inducing conditions), the effect on the T3SS is stronger than on other genes affected by CsrABC (Fig. 3a, Suppl. Table 5).”
- Line 353: Inserted that in addition to the overall molecular mechanism of CsrBC upregulation, its direct effect on VirF in *Y. enterocolitica* remains to be determined.
- Lines 399-402: Changed “This specific downregulation is conveyed by the CsrABC system via the main T3SS transcription factor VirF: Increased levels of the regulatory RNAs CsrB and especially CsrC at higher local densities sequester CsrA and thereby destabilize the *virF* transcript.” to “This specific downregulation may be conveyed by the CsrABC system via the main T3SS transcription factor VirF: Increased levels of the regulatory RNAs CsrB and especially CsrC at higher local densities sequester CsrA, which in the highly similar *Y. pseudotuberculosis* system destabilizes the *virF* transcript (Kusmierek *et al*., 2019).”
- Line 408: Changed “Influence of local higher cell density on T3SS secretion and on host-cell attachment.” to “Model of the influence of local higher cell density on T3SS secretion and on host-cell attachment.”

As indicated above, Suppl. Fig. 13 now shows the sequence conservation of the virF upstream and coding region between *Y. enterocolitica* and *Y. pseudotuberculosis*.

Part II – Major Issues: Key Experiments Required for Acceptance

Please use this section to detail the key new experiments or modifications of existing experiments that should be absolutely required to validate study conclusions.

Generally, there should be no more than 3 such required experiments or major modifications for a "Major Revision" recommendation. If more than 3 experiments are necessary to validate the study conclusions, then you are encouraged to recommend "Reject".

Reviewer #2: The authors should measure expression of the mutant reporter construct from Supplementary Figure 13 at ODin 1.5.

As stated above, we have performed this experiment with the additional control and another reporter. Given that the results show that the experiment does not directly proof the link between CsrA and VirF levels, we removed Suppl. Fig. 13 (and added a new Suppl. Fig. 13 showing the sequence similarity of the virF upstream and coding region between *Y. enterocolitica* and *Y. pseudotuberculosis*).

Part III – Minor Issues: Editorial and Data Presentation Modifications

Please use this section for editorial suggestions as well as relatively minor modifications of existing data that would enhance clarity.

Reviewer #2: Minor comment: the authors should describe Supplementary Figure 14 and their interpretation of these data in the Results section.

We moved the reference to Supplementary Figure 14, which confirms the genetic compensation of manipulations of the CsrABC system, from the legend of figure 6 to the main text (lines 254-256).
