# Supplementary material for: Yersinia actively downregulates type III secretion and adhesion at higher cell densities
Source: PLoS Pathog. 2025 Aug 12;21(8):e1013423. doi: 10.1371/journal.ppat.1013423 (PMC12404644; doi:10.1371/journal.ppat.1013423)
Supplement: Attachment — Submitted filename: Response to Main Reviewer Comments 3.docx [file ppat.1013423.s023.docx]

**Journal Requirements:**

1) Please ensure that the **CRediT author contributions** listed for every co-author are completed accurately and in full.

At this stage, the following Authors/Authors require contributions: Francesca Ermoli, Gabriele Malengo, Christoph Spahn, Timo Glatter, and Andreas Diepold. Please ensure that the full contributions of each author are acknowledged in the "Add/Edit/Remove Authors" section of our submission form.

The list of CRediT author contributions may be found here: https://journals.plos.org/plospathogens/s/authorship#loc-author-contributions

We have amended the author contributions accordingly. Please note that we added one author (as fourth author), Corentin Brianceau, who performed the additional experiments for the direct effect of CsrABC on VirF.

2) Please provide an **Author Summary**. This should appear in your manuscript between the Abstract (if applicable) and the Introduction, and should be 150-200 words long. The aim should be to make your findings accessible to a wide audience that includes both scientists and non-scientists. Sample summaries can be found on our website under Submission Guidelines:

https://journals.plos.org/plospathogens/s/submission-guidelines#loc-parts-of-a-submission

We now provide an author summary.

3) Please **upload all main figures as separate Figure files in .tif or .eps format**. For more information about how to convert and format your figure files please see our guidelines:

<https://journals.plos.org/plospathogens/s/figures>

Done.

4) We have noticed that you have uploaded Supporting Information files, but you have not included a list of legends. Please **add a full list of legends for your Supporting Information files after the references list**.

We now provide a full list of legends.

5) Some material included in your submission may be copyrighted. According to PLOSu2019s copyright policy, authors who use figures or other material (e.g., graphics, clipart, maps) from another author or copyright holder must demonstrate or obtain permission to publish this material under the Creative Commons Attribution 4.0 International (CC BY 4.0) License used by PLOS journals. Please closely review the details of PLOSu2019s copyright requirements here: PLOS Licenses and Copyright. If you need to request permissions from a copyright holder, you may use PLOS's Copyright Content Permission form.

Please respond directly to this email and provide any known details concerning your material's license terms and permissions required for reuse, even if you have not yet obtained copyright permissions or are unsure of your material's copyright compatibility. Once you have responded and addressed all other outstanding technical requirements, you may resubmit your manuscript within Editorial Manager.

Potential Copyright Issues:

- Figures 3C; Please confirm whether you drew the images / **clip-art within the figure panels** by hand. If you did not draw the images, please provide a link to the source of the images or icons and their license / terms of use; or written permission from the copyright holder to publish the images or icons under our CC BY 4.0 license. Alternatively, you may replace the images with open source alternatives. See these open source resources you may use to replace images / clip-art:

- https://commons.wikimedia.org

- https://openclipart.org/.

- The following Figure contains **screenshots**: **Supplementary File 1.** We are not permitted to publish these under our CC-BY 4.0 license, websites are usually intellectual property and are copyrighted.This includes peripheral graphics of the web browser such as icons and button. We ask that you please remove or replace it.

We have changed the clip-arts used in the final version of Fig. 3c to open-source based clip-arts. Supplementary File 1 only contains images of our custom-written script running in Fiji, an open-source platform. To the best of our understanding, this is in line with the CC-BY 4.0 license.

6) Please ensure that the **funders and grant numbers** match between the Financial Disclosure field and the Funding Information tab in your submission form. Note that the funders must be provided in the same order in both places as well.

- State the initials, alongside each funding source, of each author to receive each grant. For example: "This work was supported by the National Institutes of Health (####### to AM; ###### to CJ) and the National Science Foundation (###### to AM)."

- State what role the funders took in the study. If the funders had no role in your study, please state: "The funders had no role in study design, data collection and analysis, decision to publish, or preparation of the manuscript.".

If you did not receive any funding for this study, please simply state: u201cThe authors received no specific funding for this work.u201d

We clarified that we did not receive specific funding for this study.

Reviewer's Responses to Questions

Part I - Summary

Please use this section to discuss strengths/weaknesses of study, novelty/significance, general execution and scholarship.

**Reviewer #1**: Ermoli et al. substantially revised their manuscript and appropriately addressed my concerns. Their new finding that T3SS is confined to less dense parts of a microcolony is particularly exciting and supports the author’s model. I congratulate the authors on this impressive work and recommend publication of the manuscript.

We thank the reviewer for this very positive feedback!

**Reviewer #2**: The authors show that expression of the Yersinia enterocolitica type 3 secretion system (T3SS) is downregulated at higher cell densities. This is an interesting phenomenon that may contribute to Y. enterocolitica pathogenesis. The authors provide compelling evidence that this regulation involves control of the level/activity of VirF, a transcription activator of T3SS genes. The authors argue that VirF levels are themselves regulated by the riboregulatory CsrA/B/C system. However, their evidence that the Csr system is responsible for the observed T3SS regulation is circumstantial and not well supported by the data.

Thank you for the positive feedback. We have addressed the question of the direct influence on CsrA/B/C on VirF by performing an additional experiment, as suggested by the reviewer (see below).

**Reviewer #3**: In the present resubmitted study by Ermoli et al., the authors addressed the major points raised by the previous reviewers.

The authors convincingly showed that the Yersinia T3SS is downregulated/repressed during stationary phase and that this is not controlled by RpoS and the quorum sensing factors YenI and LsrK. In their resubmission they added that stationary phase control of T3SS is also not regulated by the stringent response RelA/SpoT. Moreover, they investigated T3SS activation in microcolonies in vitro using an sfGFP-ssrA fusion with high sensitivity and the potential for dynamic expression analyses due to the SsrA degradation tag, and found that indeed yopE expression is only induced in the growing/replicating bacteria at the surface of the colony.

The authors alternatively tested the influence of the post-transcriptional carbon starvation regulatory system CsrABC, as it was previously published to (i) influence Yersinia T3SS regulation via the control of VirF/LcrF, and (ii) vary in their expression during stationary phase in Yersinia. Based on these results and their observation that higher bacterial densities lead to higher CsrB and CsrC RNA levels, but not CsrA levels, they propose a model on CsrABC-mediated downregulation of T3SS expression.

We thank the reviewer for the positive feedback and have addressed the reminaing specific points as detailled below.

Part II – Major Issues: Key Experiments Required for Acceptance

Please use this section to detail the key new experiments or modifications of existing experiments that should be absolutely required to validate study conclusions.

Generally, there should be no more than 3 such required experiments or major modifications for a "Major Revision" recommendation. If more than 3 experiments are necessary to validate the study conclusions, then you are encouraged to recommend "Reject".

**Reviewer #1**: NA

**Reviewer #2**:

1. The authors conclude that virF expression is regulated as a function of cell density due to CsrA binding to the mRNA. However, the evidence for the involvement of CsrA is circumstantial. Figure S13B appears to address this question, but the figure panel is not mentioned in the text, and the graph is very difficult to interpret since the lines are difficult to tell apart. Mutating the CsrA binding sites in a virF translational reporter fusion would provide a more direct test of CsrA’s role.

We have adapted Fig. S13B (now Fig. S14B) to make it easier to interpret.

To directly test to influence of CsrA on VirF in the strains and conditions investigated in this manuscript, we followed the reviewer’s excellent suggestion and tested the expression of VirF in wild-type and a CsrA binding site mutant, using a reporter construct. As a binding site mutant, we used the GGA to TTC mutant of the -16 to -14 region of virF, which was found by Kusmierek *et al*. (PLOS Pathog 2019) to reduce CsrA binding. To exclude a functional influence of additional VirF, we used a truncated version retaining the complete predicted CsrA binding site, virF(-123/+75)-sfGFP. As shown in the new Suppl. Fig. 13, this mutation reduces VirF expression, in line with a direct link between CsrA and VirF levels.

2. CsrA regulates a large number of genes. If high cell density modulates CsrA activity, you would expect to see changes in the expression of other CsrA-regulated genes. The volcano plot (Figure 3A) suggests this is not the case, arguing against a model for T3SS regulation by cell density that involves CsrA. Nonetheless, it would be informative to reports changes in protein levels for known CsrA targets.

We have repeatedly observed that under T3SS-activating conditions, the effect of global regulators is less pronounced than might be expected. This may be due to the reprogramming of protein production towards T3SS components and effectors, which seems to at least partially override other regulatory pathways. We now mention this in the discussion. To directly address the reviewer request, we have analyzed the expression of all identified genes with a >4-fold transcription changed in a *csrA* mutant in *Y. pseudotuberculosis* by Bücker et al. (JBC (2014)), excluding flagellar genes, which are not expressed at 37°. As shown in the new Suppl. Table 5, these genes are clearly similarly regulated in our dataset, despite the different conditions (25° vs. 37°), but the effect is less pronounced than for the T3SS genes – again highlighting the strength and specificity of the phenotype described in the manuscript.

**Reviewer #3**: The results were obtained with quantitative fluorescence microscopy, comparative proteomic and qRT-PCR analysis etc. and have a very high quality, the density-mediated downregulation of T3SS expression is analyzed in detail and the date are very convincing.

However, the molecular mechanism how high density is sensed and transmitted to the known CsrABC-VirF-T3SS cascade and the relevance for virulence/pathogenesis or bacterial fitness remain unclear.

We thank the reviewer for the assessment. Indeed, as stated in lines 330-343 in the discussion, the factors upstream the CsrABC-VirF cascade cannot be determined from our data. While we have tested various candidates, including some initially suggested by the reviewers, none of these candidates was main to be a main contributing factor, similarly to what has been the case in previous studies of CsrABC-related phenotypes. In addition to the extensive discussion in lines 330-342, we now specifically reiterate this point in lines 343-344.

Part III – Minor Issues: Editorial and Data Presentation Modifications

Please use this section for editorial suggestions as well as relatively minor modifications of existing data that would enhance clarity.

**Reviewer #1**: NA

**Reviewer #2**:

1. Figure 3A. It would be helpful to indicate VirF on the volcano plot.

As VirF has not been introduced at this point, we feel that it would be disruptive and potentially confusing to highlight VirF in this figure. However, we have now specifically indicated it in Suppl. Fig. 6, the volcano plot showing the different T3SS categories, and refer to this figure in the respective part of the results section (lines 205-207).

2. Line 209. Overexpressing virF restores T3SS expression at higher cell densities. The authors conclude that T3SS repression at higher cell densities is because of reduced virF expression, but it could be due to reduced VirF activity or activity of a repressive factor that counteracts the effects of VirF.

To indicate this possibility, we replaced “…is a consequence of reduced VirF expression” by “…VirF activity”.

3. Line 230. “Indicate” is too strong a conclusion here. “Suggest” would be more appropriate. VirF translation or protein stability could be the regulated process, or there could be a promoter immediately upstream of virF.

We thank the reviewer for this suggestion and have changed the word accordingly.

4. Line 228. While SctG levels are less affected than those of VirF, it would be helpful for the authors to mention here that SctG levels are lower at the higher cell density.

We now explicitly mention this: “Total proteome analysis and immunoblots showed that, while SctG is affected by higher cell densities, the effect is significantly weaker than for VirF or other T3SS components”

5. Line 231-2. “To discriminate between transcriptional and post-transcriptional regulation”. Measuring RNA level does not distinguish between regulation of transcription and regulation of RNA stability.

Based on the available data, sctG and virF are transcribed together, but regulated differently after transcription, with the latter point being influenced by binding of CsrA to the transcript. To make this clear, we now more specifically mention this reasoning at this point: “To discriminate between regulation of transcription of *sctG-virF* and post-transcriptional regulation specifically affecting *virF…”*

**Reviewer #3**:

lines 155-159

This interpretation is unclear to me “secretion was neither suppressed at low density by spent medium”. In Fig. 3c the fluorescence of the PyopE-sfgfp-ssrA reporter is decreased (P<0.0001) after media change. Please explain.

We now specifically mention this finding and briefly discuss possible reasons for the reduction of secretion after addition of spent medium. We also took the change to simplify the respective part of the results part. The respective part (line 170-173 in the revised manuscript was changed to: “Secretion at low bacterial density was consistently slightly reduced (by 30-40%) by the addition of spent medium from high-density cultures, indicating a possible effect of nutrient limitation or changed pH. In contrast, secretion was not activated at high densities by provision of fresh medium (Fig. 3c).“ We hope that this makes the interpretation clearer.
